# The combined efficacy of OTS964 and temozolomide for reducing the size of power-law coded heterogeneous glioma stem cell populations

**DOI:** 10.18632/oncotarget.26800

**Published:** 2019-03-22

**Authors:** Michiya Sugimori, Yumiko Hayakawa, Ryoi Tamura, Satoshi Kuroda

**Affiliations:** ^1^ Department of Integrative Neuroscience, University of Toyama, Toyama, Toyama 930-0194, Japan; ^2^ Department of Neurosurgery, University of Toyama, Toyama, Toyama 930-0194, Japan

**Keywords:** glioma stem cell (GSC), glioma sphere (GS), OTS964, temozolomide, population size

## Abstract

Glioblastoma resists chemotherapy then recurs as a fatal space-occupying lesion. To improve the prognosis, the issues of chemoresistance and tumor size should be addressed. Glioma stem cell (GSC) populations, a heterogeneous power-law coded population in glioblastoma, are believed to be responsible for the recurrence and progressive expansion of tumors. Thus, we propose a therapeutic strategy of reducing the initial size and controlling the regrowth of GSC populations which directly facilitates initial and long-term control of glioblastoma recurrence. In this study, we administered an anti-glioma/GSC drug temozolomide (TMZ) and OTS964, an inhibitor for T-Lak cell originated protein kinase, in combination (T&O), investigating whether together they efficiently and substantially shrink the initial size of power-law coded GSC populations and slow the long-term re-growth of drug-resistant GSC populations. We employed a detailed quantitative approach using clonal glioma sphere (GS) cultures, measuring sphere survivability and changes to growth during the self-renewal. T&O eliminated self-renewing GS clones and suppressed their growth. We also addressed whether T&O reduced the size of self-renewed GS populations. T&O quickly reduced the size of GS populations via efficient elimination of GS clones. The growth of the surviving T&O-resistant GS populations was continuously disturbed, leading to substantial long-term shrinkage of the self-renewed GS populations. Thus, T&O reduced the initial size of GS populations and suppressed their later regrowth. A combination therapy of TMZ and OTS964 would represent a novel therapeutic paradigm with the potential for long-term control of glioblastoma recurrence via immediate and sustained shrinkage of power-law coded heterogeneous GSC populations.

## INTRODUCTION

Cancer stealthily occurs from initiating cells [1–5] which continuously reproduce and plastically generate diverse cell types, leading to construction of an extensive heterogeneous cell population [5–10]. The cell population survives and reproduces over generations while recapitulating its heterogeneity through self-renewal of heterogeneous cell populations [7, 11–15]. Thus, when cancerous tumors are found in clinics they should be treated as abnormal tissue where heterogeneous initiating cell populations reside [16].

Glioblastoma is a primary malignant brain tumor that resists chemoradiotherapy then recurs to become a fatal space-occupying lesion [17–20]. Issues in glioblastoma chemoradioresistance and tumor size should be resolved to improve the prognosis of patients [14, 17, 19–23]. The initiating cells in glioblastoma, glioma stem cells (GSCs), are believed to be responsible for tumor recurrence after chemoradiotherapy and for progressive expansion of tumor size [4, 5, 14, 19, 23–30]. Thus, GSCs should be targeted to control glioblastoma recurrence and tumor size [19, 28, 30, 31], although how they should be targeted remains unclear.

In seeking to evaluate the efficacy of potentially GSC-targeting drugs, to address the dual issues of recurrence and regrowth we have employed a quantitative approach to assess survivability and regrowth using clonal tumor neurosphere cultures. This facilitates constructing a model of GSC clonal tumor neurosphere populations that quantifies GSC chemoradiotherapy survivability, the size of surviving clones following chemoradiotherapy, and recurring chemoradioresistant GSC population size [11, 14, 30]. We concluded that GSCs are heterogeneous in their growth properties and chemosensitivity, proposing that heterogeneous GSC populations reproduce themselves via their plasticity: “self-renewal of the GSC populations” [8, 11, 14, 23, 27]. Moreover, we found that GSCs are plastically able to acquire chemoresistance [14]. We conclude that this experimental method can facilitate evaluating novel therapies where GSCs are targeted by tracking changes to survivability, regrowth rates, and recurrent population size. The survival and growth rates of GSCs administered chemoradiotherapy yield essential information for establishing a novel therapeutic strategy that involves reducing the initial size and suppressing the regrowth of robust heterogeneous GSC populations. This strategy could directly facilitate initial and long-term control of glioblastoma recurrence [11, 14].

We previously discovered that GSC populations are heterogeneous and their growth is coded by a scale-free power-law [11]. The power-law represents a spatiotemporal recapitulation of GSC population heterogeneity through continuous population growth. We proposed a hypothetical strategy where a disruption of the power-law may eliminate the scale-free power-law coded heterogeneous GSC populations, facilitating long-term control of glioblastoma recurrence. We also found that power-law growth was not disrupted by the glioblastoma/GSC agent “temozolomide (TMZ)” or an inhibitor for T-Lak cell originated protein kinase (TOPK), OTS964, although both TMZ and OTS964 affected GSC population growth in different ways [11, 14, 22, 32–35]. These studies also showed that following exposure to TMZ and OTS964, resistant GSC clones survived to recapitulate power-law growth, suggesting heterogeneous power-law coded GSC populations are quite robust. Thus, we showed that TMZ or OTS964 alone do not induce extinction in heterogeneous GSC populations because of their robustness [29]. Because the strategy of inducing GSC population extinction was unsuccessful, another potential growth disruption strategy would be to first shrink the size of GSC populations as much as possible then suppress their regrowth [14], which could potentially inhibit long-term glioblastoma recurrence.

In this study, we administered TMZ and OTS964 in combination [30, 36–42] using the clonal tumor neurosphere culture approach (explained above), investigating whether together they more successfully and substantially shrank the initial size of power-law coded GSC populations and whether they slowed the long-term regrowth of drug-resistant GSC populations [15].

## RESULTS

### OTS964-administered and resistant GS clones survived long-term and grew to recover their populations

**Figure 1:**
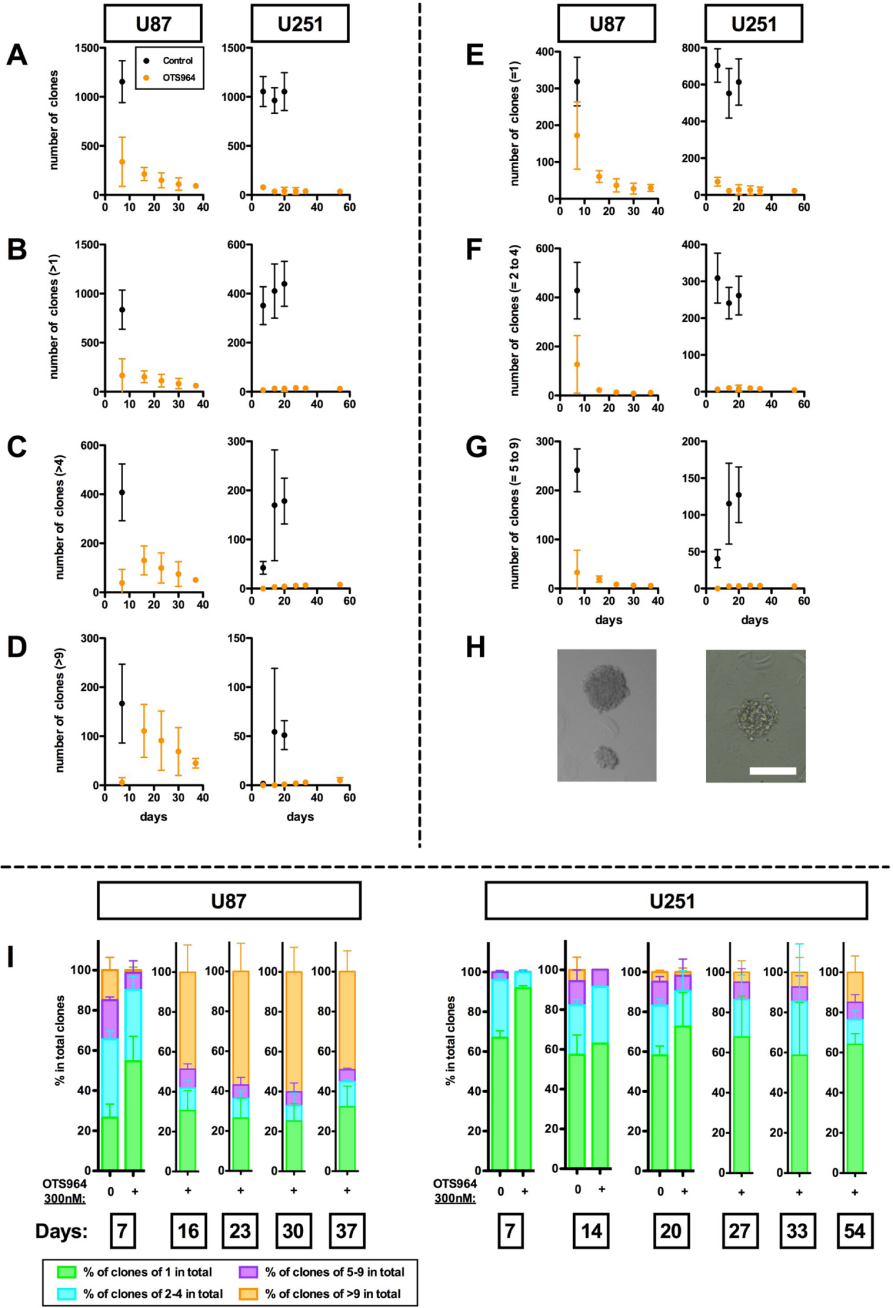
Long-term survival of OTS964-resistant GS clones We seeded U87- and U251-glioma cells in methylcellulose-containing growth medium at an uniform clonal density. Each clone differentially grew with some becoming glioma spheres: GSs. The data are derived from assays at days 7, 16, 23, 30 and 37 for U87-GS clones and at days 7, 14, 20, 27, 33 and 54 for U251-GS clones in 300 nM of OTS964 administered experiments; in control experiments for U87-GS clones at day 7 and for U251-GS clones at days 7, 14 and 20. (**A**–**G**) The graphs show the number of total clones (A); multi-cellular clones (>1) (B); clones with more than 4 cells (>4) (C); large clones with more than 9 cells (>9) (D); single-cell clones (=1) (E); clones with 2 to 4 cells (=2 to 4) (F); and clones with 5 to 9 cells (=5 to 9) (G). The total number of surviving OTS964-administered GS clones gradually decreased week by week, while a significant number of large clones appeared in later weeks. (**H**) The pictures are self-renewed and expanded GS clones administered 300 nM of OTS964 for 16 and 54 days in U87- and U251-GS clones, respectively. The scale bar indicates 200 μm intervals. (**I**) Increase in the percentages of large OTS964-resistant GS clones in the later weeks. The graph bars show the size of surviving OTS964- resistant GS clones. While the total surviving OTS964-resistant GS clones decreased, the percentage of large GS clones increased in later weeks.

We previously showed that an inhibitor for T-LAK cell originated protein kinase (TOPK), OTS964, reduced the size of glioma stem cell (GSC) populations (GSC population cell numbers) as represented by glioma sphere (GS) populations in two ways; through clone elimination and through disturbing clone growth in a dose dependent manner [14, 32]. Application of 300 nM of OTS964 significantly reduced the number of U87-derived and U251-derived self-renewing GS clones (U87- and U251-GS clones, respectively) to about 30% at day 7 for U87- and to <10% after day 7 for U251-GS populations when compared to controls (Figure 1A; control shown in black and OTS964 in orange). The number of surviving single-cell (=1) clones was relatively high, while multi-cellular GS clones were strongly reduced at day 7, suggesting that OTS964 suppressed the self-renewal of GS clones (Figure 1B–1G). The percentage of single-cell clones was consistently high in the OTS964-administered GS clones at day 7. We thus reproduced the clone-eliminating and growth-disturbing efficacies of OTS964 in the GS populations.

We previously reported that OTS964-administered GS populations recover to the size of control GS populations in following generations [14]. However, we have not traced how and whether surviving OTS964-administered GS clones grow for more than one week, and so in this investigation we traced the number and size of GS clones for about one month. The number of OTS964-administered GS clones gradually decreased throughout the period, with about ninety U87- and about forty U251-GS clones surviving after one month (Figure 1A). We surprisingly found that a significant percentage of the GS clones expanded to more than 10 cells (Figure 1D, 1H and 1I). It appears that the size of the surviving GS clones grew starting from day 16, leading to proportional expansion of the U87-GS population size (Supplementary Figure 1). About half of the surviving OTS964-administered U87- and more than 10 percent of U251-GS clones contained more than 10 cells, which we classified as large GS clones. Most of these large clones expanded considerably by the end of the month to contain hundreds of cells (Figure 1H, pictures). This suggests that a significant proportion of the surviving OTS964-administered GS clones considerably expanded and were responsible for the proportional expansion of the OTS964-administered GS populations.

### Surviving long-term OTS964-resistant GS clones maintained power-law growth

**Figure 2:**
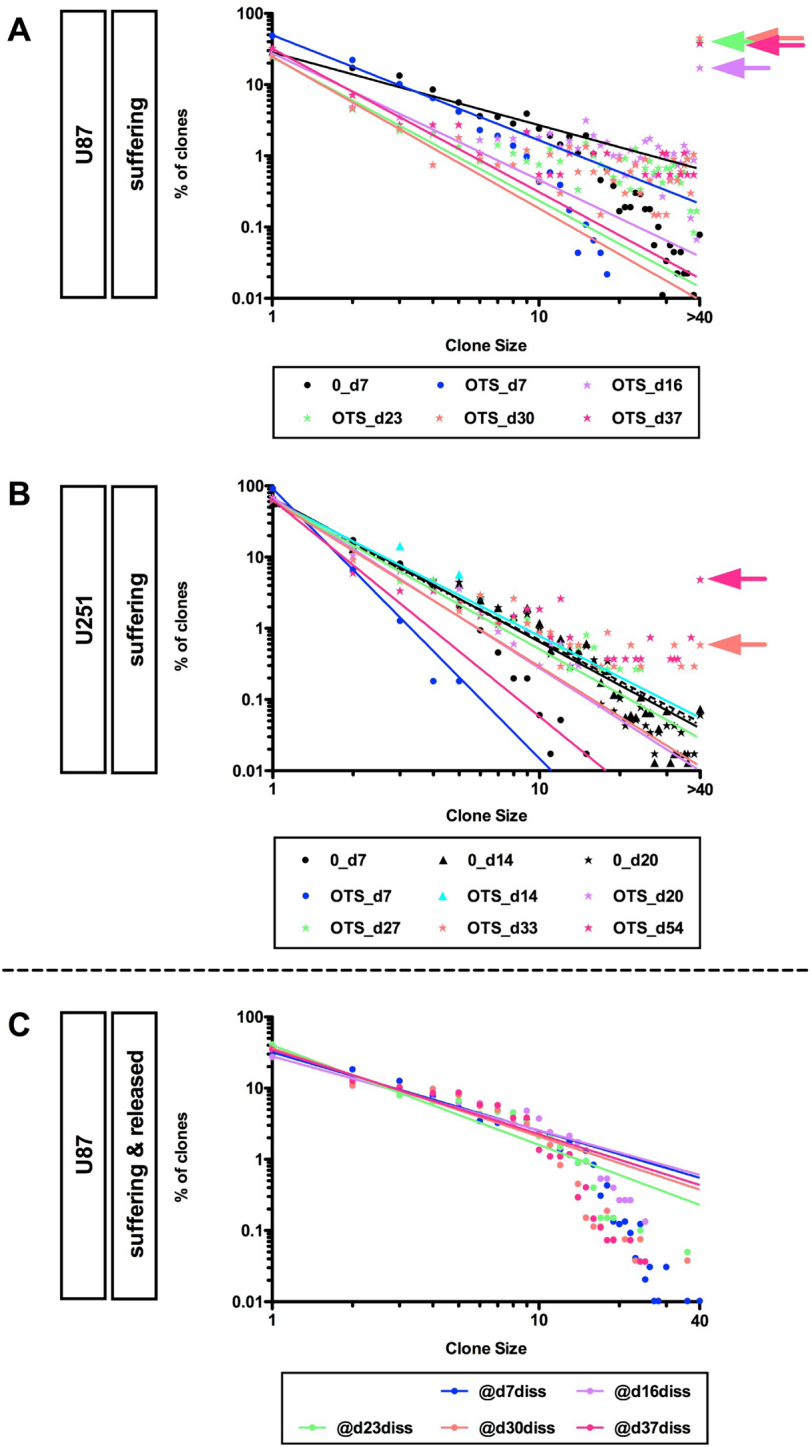
Recapitulation of diversity and power-law growth of surviving OTS964-administered and recovered GS populations (**A** and **B**) Maintenance of the power-law growth in the surviving OTS964-administered “growing” GS populations. The graphs show double logarithmic plots of the clone size (number of cells in each clone) and the frequency of U87- and U251-GS clones at day 7 for U87-, and at days 7, 14 and 20 for U251-GS clones for the control; at days 7, 16, 23, 30 and 37 for surviving OTS964-administered U87-, and days 7, 14, 20, 27, 33 and 54 for surviving OTS964-administered U-251 GS clones. From days 16, 23, 30 and 37 for the U87-GS clones and from days 33 and 54 for the U251-GS clones GS clones with more than 40 cells were assigned a value of 40 because we unable to practically quantify the size of such large clones. Thus, the regression lines are derived from the data for 1-cell to 39-cell GS clones (excluding the 40-cell GS clone data, then regression lines were acquired for the double logarithmic plots: see the arrows for the >40 cell plots). (**C**) Recapitulation of diversity and power-law growth in the recovered OTS964-administered U87-GS populations. All the graphs show that a power-law was recapitulated in the recovered OTS964-administered GS populations after passage and dissociation from surviving OTS964-administered GS clones at days 7, 16, 23, 30 and 37 (@d7diss, @d16diss, @d23diss, @d30diss and @d37diss, respectively), even though major fractions of the cells were derived from large GS clones in the experiments from days 16, 23, 30 and 37 (see Figure 1; Supplementary Figures 1, 2).

OTS964-resistant GS clones survived long-term and expanded considerably, suggesting that the expandable OTS964-resistant GS clones were selectively enriched (Figure 2A and 2B; percentages of clones with 40 or more cells are 17.1%, 41.0%, 45.0% and 37.7% at days 16, 23, 30 and 37 for U87-GS clones; 0.6% and 4.9% at days 33 and 54 for U251-GS clones, respectively). We then asked whether the long-term surviving OTS964-resistant GS populations continued to follow a power-law. Because we were not able to quantify the number of cells in clones with more than 40 cells, we first asked whether GS clones with less than 40 cells followed a power-law, excluding clones with 40 or more cells from the data and applying double logarithmic regression lines. Surprisingly, both the U87- and U251-GS populations followed the power-law in GS clones with less than 40 cells (Figure 2A and 2B; Supplementary Figure 2A). This suggests that the OTS964-resistant GS populations maintain heterogeneity as they exhibit power-law growth in the relatively less populous (<40 cell) GS clones.

### GS clones derived from the expanded OTS964-survived/resisted GS clones exhibited a power-law in their growth recovery

We next asked whether the expandable OTS964-resistant GS clones maintain power-law growth properties as they repopulate [14] (Supplementary Figure 2B). Because the expandable OTS964-resistant GS clones were selectively enriched, it appears that the expandable OTS964-resistant GS clones may have lost their power-law growth properties. The enriched expandable OTS964-resistant U87-GS clones were progressively responsible for GS population size increases. Thus, the cells derived from the selected U87-GS clones predominantly comprised the expanded GS populations. We found that every U87-GS population derived from long-term survived OTS964-resisted GS populations in days 16, 23, 30 and 37 closely followed a power-law (Figure 2C; Supplementary Figure 2B), suggesting that expandable OTS964-resistant GS clones never lost or had their power-law growth properties disrupted. Thus, surviving OTS964-resistant GS clones continuously grew and progressively expanded their populations while maintaining heterogeneity and exhibiting power-law growth.

### Temozolomide suppressed self-renewing GS population growth via eliminating clones and disturbing clone growth

**Figure 3:**
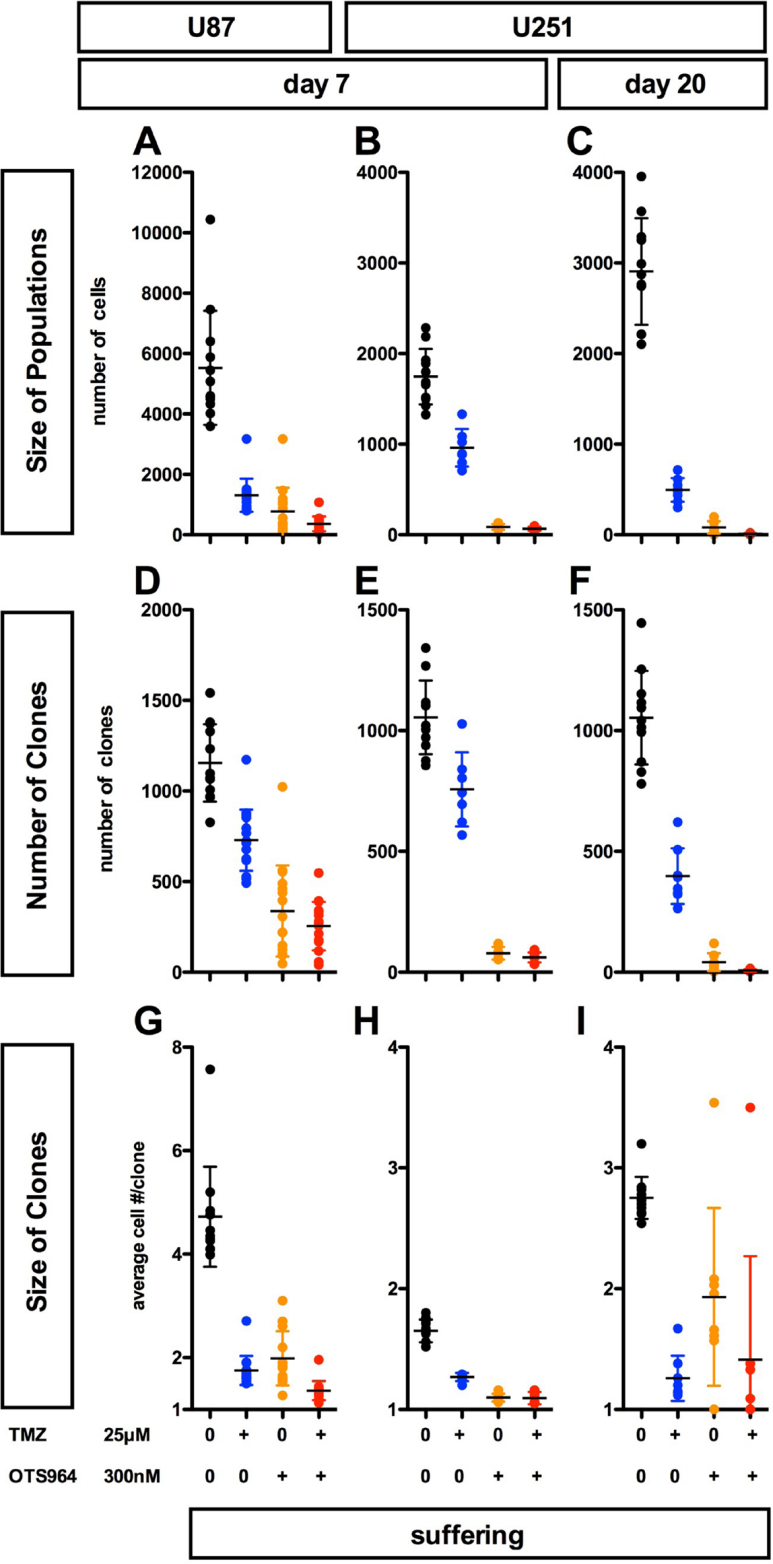
Complementary suppression of GS population expansion with OTS964 and TMZ; combined efficacy of OTS964 and TMZ in self-renewing GS populations' shrinkage (**A**–**I**) The graphs show the size of GS populations (A–C), the number of GS clones (D–F) and the size of GS clones (G–I) in the U87-GS populations at day 7 (A, D and G), and in the U251-GS populations at days 7 (B, E and H) and 20 (C, F and I). 300 nM of OTS964 reduced GS populations via elimination of GS clones and suppression of the growth of GS clones (see the orange circles), while 25 μM of temozolomide (TMZ) also reduced the size of the GS populations via significant suppression of GS clone growth and survival (see the blue circles). However, the clone-eliminating activity of TMZ was smaller than that of OTS964 (A–I; see also Sugimori *et al*, 2015; 2018). The combined efficacy of OTS964 and TMZ (T&O) was stronger than each single administration of OTS964 or TMZ for both eliminating clones and disturbing growth (see the red circles). Statistical analyses of the graphs are included in Supplementary Figure 3.

We previously reported resistance to clone elimination in sequential administration of OTS964 [14]. The appearance of expandable OTS964-resistant GS clones following long-term administration (Figures 1 and 2) suggests that continuous administration of OTS964 alone cannot suppress GSC population size. We also reported that temozolomide (TMZ) suppresses U87-GS clone growth in a dose dependent manner although a significant fraction of GS clones survive [11]. However, little is known about the growth of TMZ-resistant heterogeneous GS populations. We first addressed whether and how TMZ alone shrinks the size of self-renewing GS populations. We previously reported that 25 μM of TMZ eliminated U87-GS clones as well as 125 and 625 μM of TMZ, and 125 and 625 μM of TMZ inhibited the growth of heterogeneous U87-GS populations, suggesting that TMZ clone elimination was not dose dependent [11, 14]. Moreover, 25 μM of TMZ suppressed U87-GS clone growth, resulting in GS populations with smaller U87-GS clones [11]. We thus determined that 25 μM of TMZ was appropriate for the further experiments described here. 25 μM of TMZ sufficiently reduced the size of both U87- and U251-GS populations (Figure 3A–3C, see blue circles; Supplementary Figure 3A) and suppressed the growth of GS clones, although a significant proportion of GS clones survived (Figure 3D–3I, see blue circles; Supplementary Figure 3B and 3C). Thus, TMZ shrinks the size of GS populations via clone elimination and continuous suppression of the growth of GS clones.

**Figure 4:**
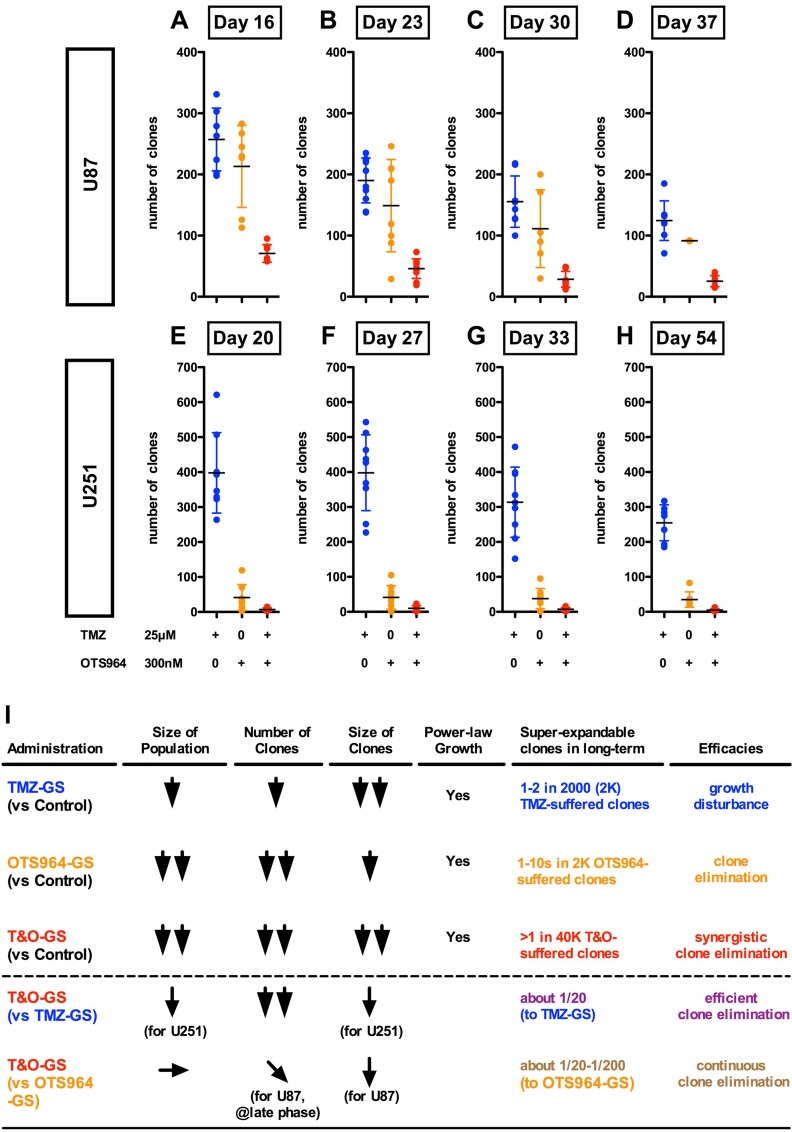
Substantial elimination of GS clones in combined administration of TMZ and OTS964 (**A**–**H**) The graphs show the number of U87-GS clones in the different administration paradigms of TMZ (blue circles), OTS964 (orange circles) and in combination (T&O; red circles) at days 16 (A), 23 (B), 30 (C) and 37 (D), and the number of U251-GS clones at days 20 (E), 27 (F), 33 (G) and 54 (H). Statistical analyses of the graphs are included in Supplementary Figure 4. (I) The table shows the summary for TMZ, OTS964 and T&O efficacy for inhibiting the self-renewal of heterogeneous GS populations.

300 nM of OTS964 also shrinks the size of U87- and U251-GS populations via clone elimination and disturbing growth [14] (Figure 3, see orange circles). OTS964 eliminated significantly more GS clones when compared to TMZ (Figure 3D–3F; Supplementary Figure 3B). The appearance of expandable OTS964-resistant GS clones in long-term administration suggests that its ability to disturb growth is time-limited. It appears that the size-reducing properties of TMZ and OTS964 in GS population self-renewal are complementary with respect to clone elimination and growth disturbance (see Figure 4I).

### Administration of TMZ and OTS964 in combination substantially suppressed GS population growth via efficient clone elimination and growth disturbance

We hypothesized that the combined administration of TMZ and OTS964 (T&O) would suppress the long-term growth of self-renewing GS populations. T&O significantly reduced the size of self-renewing GS populations at days 7 and 20 for U87- and U251-GS populations (Figure 3A–3C, see red circles; Supplementary Figure 3A). T&O significantly eliminated more GS clones compared to TMZ alone (Figure 3D–3F; Supplementary Figure 3B) and strongly disturbed the growth of U87-GS clones compared to OTS964 alone (Figure 3G–3I; Supplementary Figure 3C). The two compounds in combination thus significantly reduced U251-GS population size compared to TMZ alone (Figure 3B and 3C; Supplementary Figure 3A). This suggests that combined administration of TMZ and OTS964 is superior to TMZ or OTS964 alone in reducing self-renewing GS populations via a combination of eliminating clones and disturbing their growth (see Figure 4I).

### Combined administration of TMZ and OTS964 suppressed long-term GS clone survival

As described above, T&O is superior to TMZ or OTS964 alone in reducing self-renewing GS population size via a combination of eliminating clones and disturbing growth (Figure 3). Additionally, OTS964-administered GS clones survived long-term, expanding continuously to increase the GS population size (Figure 1; Supplementary Figure 1). We next asked whether T&O-exposed GS clones regenerate the GS population size. We first followed the long-term survival of drug-administered GS clones. We found a significant reduction in the number of surviving T&O-administered U87- and U251-GS clones compared to surviving TMZ- and OTS964-administered GS clones (Figure 4A–4H; Supplementary Figure 4). This suggests that T&O efficiently eliminated GS clones that could have become resistant to independent administration of TMZ or OTS964. We did not find growing GS clones with more than 5 cells in subsequent days in the surviving T&O-exposed GS clones, while some TMZ-exposed GS clones grew considerably, with hundreds of cells in both the U87- and U251-GS populations. This suggests that T&O substantially reduced the long-term size of GS populations by disturbing the self-renewal of heterogeneous GS populations via consistent elimination of GS clones and relatively stable suppression of clone growth. Thus, combined administration of TMZ and OTS964 may be able to control GS population size, consistent with long-term recurrence control (also see text in Supplementary Materials and Supplementary Figures 5–7 regarding resistant phenotype survival of T&O-administered GS clones).

### Combined administration of TMZ and OTS964 efficiently reduced the size of self-renewed GS populations

**Figure 5:**
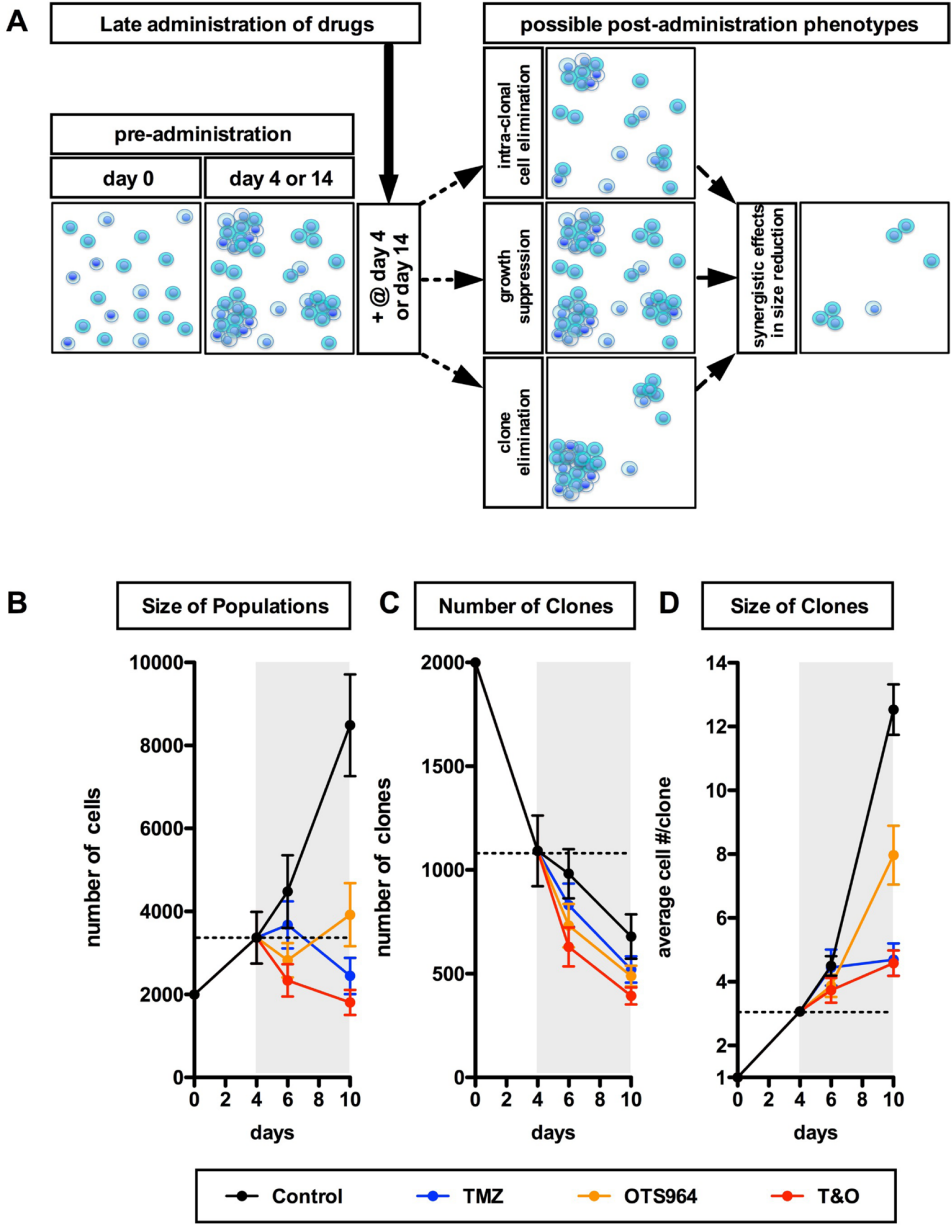
Early and continuous reduction in the size of self-renewed U87-GS populations through combined administration of TMZ and OTS964 (**A**) A schematic diagram for the late administration paradigms for the expanded and self-renewing GS populations and for possible phenotypes resulting from GS population size reduction following late administration. (**B**–**D**) The graphs show sequential changes in the size of U87-GS populations (B), the number of GS clones (C) and the size of GS clones (D) for 4 different administration paradigms (control shown in black circles; TMZ in blue; OTS964 in orange; combined TMZ and OTS964: T&O in red) from day 0 to 10. The data from day 4 are equivalent to the pre-administration status for days 6 and 10 (2 and 6 days following administration). The gray backgrounds indicate the term of drug administration. The dotted lines indicate the average of the pre-administration data at day 4. The data show mean ± standard deviation. Statistical analyses for the graphs are included in Supplementary Figure 8.

**Figure 6:**
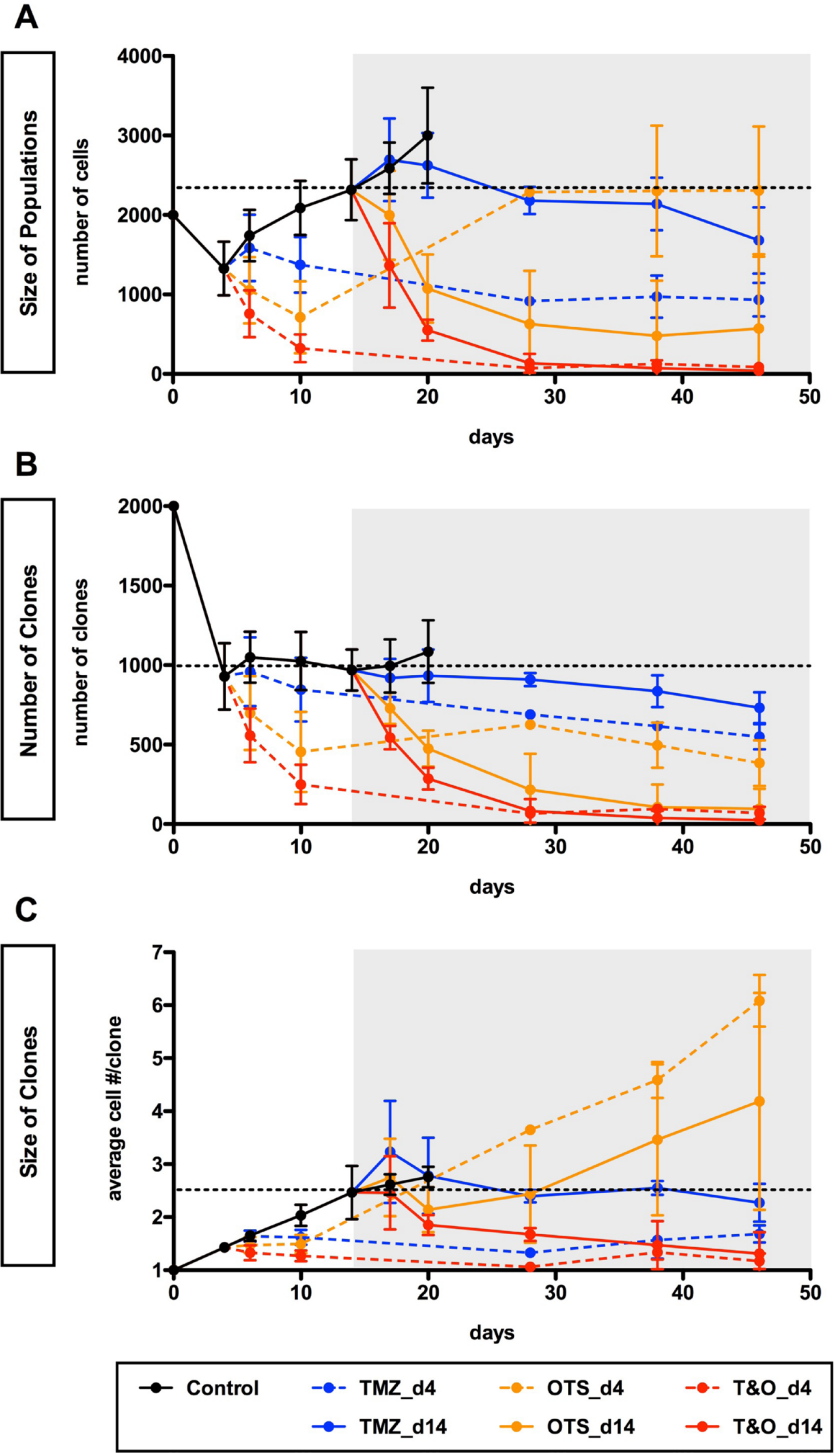
Early and long-term control of self-renewed U251-GS populations through combined administration of TMZ and OTS964 (**A**–**C**) The graphs show sequential changes in the size of U251-GS populations (A), the number of GS clones (B) and the size of GS clones (C) in the 7 different administration paradigms (control shown in black circles and solid line; TMZ in blue; OTS964 in orange; combined TMZ and OTS964: T&O in red; drug-administration at day 4 and 14 is shown with dashed and solid lines, respectively) from day 0 to 46. The data from day 4 are equivalent to the pre-administration status for days 6 and 10 (2 and 6 days after the administration, respectively); day 14 is as the pre-administration status for days 17 and 20 (3 and 6 days after the administration, respectively). The gray backgrounds indicate the term in which U251-GS clones had been administered the drugs since day 14. The dotted lines indicate the average of the pre-administration data at day 14. The data show mean ± standard deviation. The statistical analyses for the graphs are shown in Supplementary Figures 9 to 11.

We showed that T&O suppressed the self-renewal of GS populations via efficient elimination and stable suppression of the growth of GS clones. However, in patients at the time of diagnosis GSC populations would have already expanded. Thus, a remaining question is whether T&O administration shrinks “self-renewed” GSC population size. We grew and expanded the GS populations then administered T&O to the self-renewed GS populations at day 4 for U87- and days 4 and 14 for U251-GS populations. Thus, the drug-administered self-renewed and expanded GS populations could have started to shrink from day 4 or 14 via cell or clone elimination and/or growth suppression (Figure 5A). We then quantified the size and number of GS clones at 2 and 6 days following T&O administration (at days 6 and 10, respectively) for U87-GS populations; at 2, 6 and later days for “day 4 experiments”, and at 3, 6 (at days 17 and 20, respectively) and later days for the “day 14 experiments” following T&O administration for U251-GS populations. T&O significantly shrank the self-renewed GS populations 2 or 3 days after T&O administration (Figures 5B and 6A; Supplementary Figures 8A and 9), suggesting that T&O immediately reduced the self-renewed GS population size. All the TMZ-, OTS964- and T&O-administered GS populations showed a reduction in the number of GS clones; the T&O-administered GS populations showed immediate and the most efficient elimination of GS clones 2 or 3 days following administration, especially significant compared to TMZ alone (Figures 5C and 6B; Supplementary Figures 8B and 10). Moreover, the growth of T&O-administered GS clones was significantly suppressed soon after administration (Figures 5D and 6C; Supplementary Figures 8C and 11). This suggests that T&O immediately reduced the self-renewed GS population size via efficient clone elimination and immediate suppression of GS clone growth.

OTS964-administered GS populations appeared to start to shrink self-renewed GS population size following administration via clone elimination and mild suppression of clone growth (Figures 5 and 6; see orange circles; Supplementary Figures 8–11). However, OTS964-administered GS clones gradually overcame the disturbance of growth in both U87- and U251-GS populations, with the size of the populations eventually recovering (Figures 5 and 6; Supplementary Figures 8–10) through recapitulated clonal expansion of the surviving OTS964-resistant clones (see Figures 1 and 2; Supplementary Figures 1, 2, 5, 6 and 7; see text in Supplementary Materials) [14]. This again suggests that a simple administration paradigm of OTS964 alone is insufficient for long-term control of GS populations due to unstable growth disturbance.

While OTS964- and T&O-administered GS populations tended to show immediate clone elimination and growth disturbance, TMZ-administered GS populations did not show significant clone elimination compared to control (Figures 5C, 5D, 6B and 6C; see blue circles; Supplementary Figures 8, 10 and 11). However, TMZ-administered GS clones showed rather slow-starting growth disturbance, associated with growth suppression in GS population size over time, suggesting that TMZ strongly suppressed GS population growth for the long term.

T&O-administered self-renewed GS population size was continuously reduced due to substantial clone elimination and stable growth suppression, suggesting long-term control of GS population regrowth by T&O (Figures 5 and 6; see red circles at days 6, 10, 17, 20 and later days in Supplementary Figures 8–11). Thus, T&O was superior to TMZ or OTS964 alone in immediate and continuous reduction of “self-renewed/expanded” GS population size via efficient clone elimination and immediate and stable growth disturbance (See Figure 8).

### The power-law growth of self-renewed GS populations was not disrupted by TMZ and/or OTS964 administration

**Figure 7:**
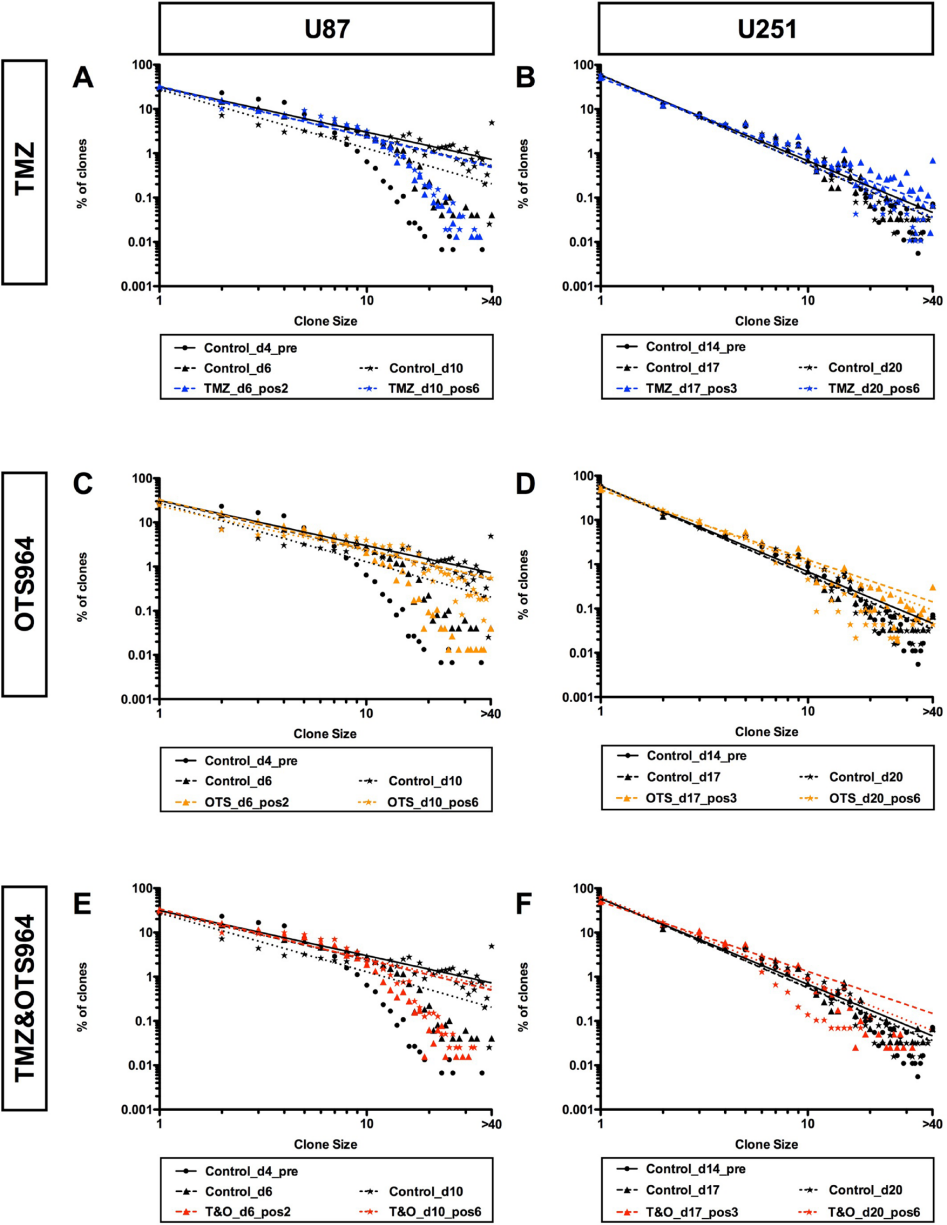
Self-renewed and expanding GS populations maintain power-law coded heterogeneity with combined TMZ and OTS964 administration despite early and continuous shrinkage (**A**–**F**) The graphs show double logarithmic plots of the clone size (number of cells in each clone) and the frequency of U87- (A, C and E) and U251-GS clones (B, D and F) in different late administration paradigms of 25 μM of TMZ (A and B, shown in blue), 300 nM of OTS964 (C and D, shown in orange), and the combined administration of TMZ and OTS964 (E and F; T&O, shown in red). Graphs A, C and E show controls (shown in black) at day 4 for the pre-administration (Control_d4_pre), and control populations at days 6 and 10 (Control_d6 and Control_d10) for 2 and 6 days following administration, respectively. Graphs B, D and F show controls (shown in black labels) at day 14 for the pre-administration (Control_d14_pre), and control populations at days 17 and 20 (Control_d17 and Control_d20) for 3 and 6 days following administration, respectively. Circles, triangles and stars denote data at days 4, 6 and 10 for U87-GS clones and days 14, 17 and 20 for U251-GS clones, respectively. The double logarithmic regression lines are shown in solid-, dashed-, and dotted lines for the frequency distributions at days 4, 6 and 10 for U87-GS clones and days 14, 17 and 20 for U251-GS clones, respectively.

**Figure 8:**
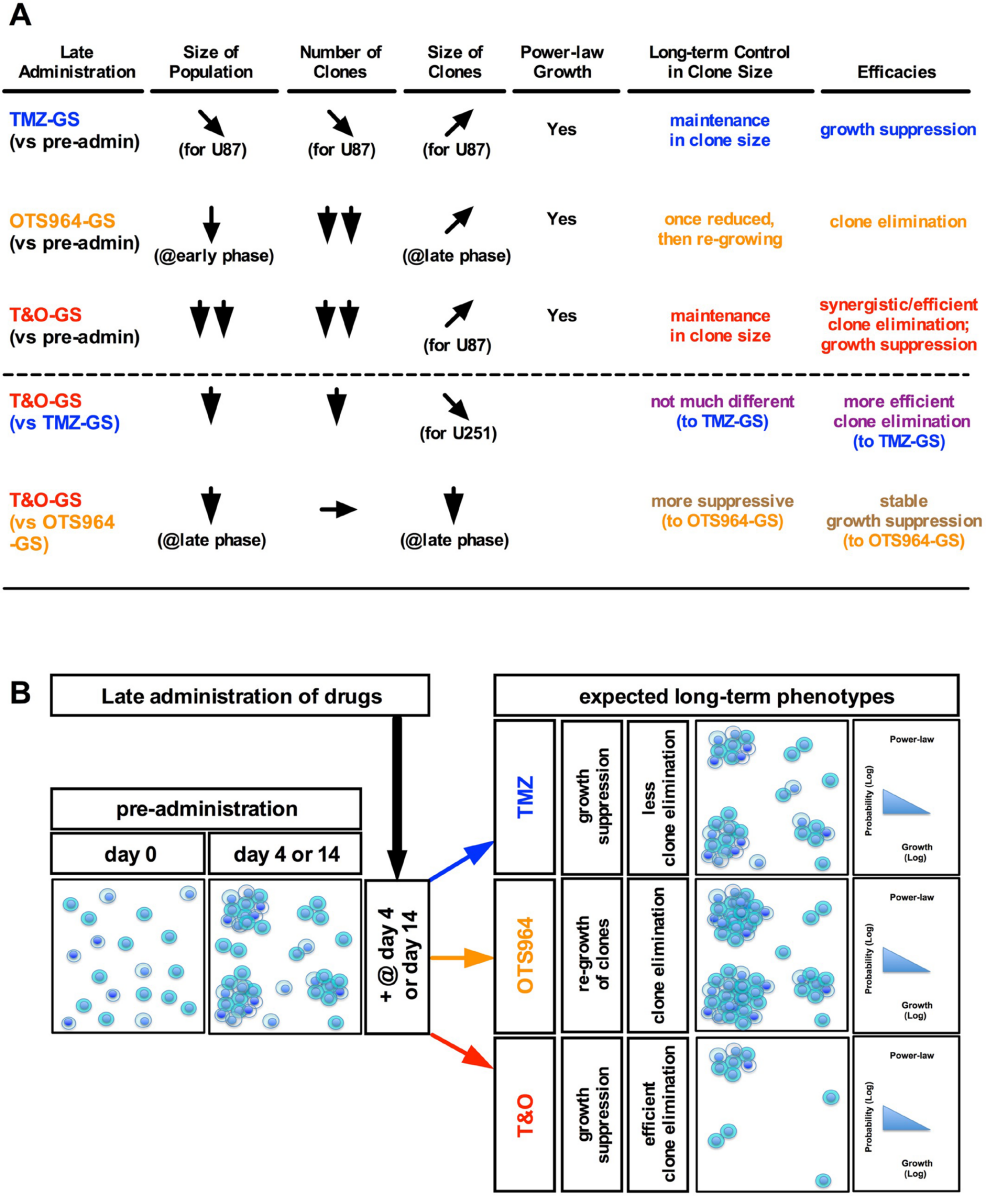
Combined administration of TMZ and OTS964 shrank GS population size via efficient clone elimination and clone growth suppression (**A**) The table shows the summary for TMZ-, OTS964-, and T&O-administered GS populations (TMZ-GS, OTS964-GS and T&O-GS) in the shrinkage of self-renewed and expanding heterogeneous GS populations. (**B**) Shows a schematic diagram of expected long-term phenotypes in the shrinkage of self-renewed and expanding heterogeneous GSC populations following late administration.

We next asked whether the power-law growth of self-renewed GS populations was disrupted following administration of TMZ and/or OTS964 and GS population size reduction. We first confirmed the GS populations exhibited power-law growth preceding administration (Figure 7, see black circles; Supplementary Figure 12), which they did for both U87- and U251-GS populations, with the control GS populations maintaining power-law growth (Figure 7; see the solid, the dashed and the dotted lines for days 4, 6 and 10 for U87-GS populations and for days 14, 17 and 20 for U251-GS populations; the regression lines were derived using the black circles, triangles and stars for days 4, 6 and 10 for the U87-GS populations and for days 14, 17 and 20 for the U251-GS populations, respectively). Thus, the self-renewed GS populations maintained power-law growth continuously. We then asked whether the power-law growth of the self-renewed GS populations was disrupted following TMZ and/or OTS964 administration. The frequency distributions of TMZ-, OTS964- and T&O-administered GS populations followed a power-law growth (Figure 7; see the circles shown in blue, orange and red for TMZ-, OTS964- and T&O-administered GS populations, respectively). Surprisingly, the regression lines for the TMZ-, OTS964- and T&O-administered GS populations tightly followed the control lines (Figure 7; see the dashed and the dotted lines drawn in blue, orange and red for the TMZ-, OTS964- and T&O-administered GS populations, respectively). This suggests that the self-renewed GS populations' power-law growth was not disrupted by TMZ and/or OTS964 administration. Thus, even though the TMZ-, OTS964- and T&O-administered GS populations exhibited a reduction in self-renewed/expanded GS population size, we conjecture that their heterogeneity was preserved during shrinkage for each of the drug-administered populations (Figure 8).

## DISCUSSION

### The distinct and combined efficacies of TMZ and OTS964 in reducing glioma stem cell population size

We previously reported that inhibition of TOPK by OTS964 substantially reduced glioma stem cell population size via eliminating clones and disturbing growth [14]. We furthermore proposed a size-reducing therapy using OTS964 to control glioblastoma recurrence [14]. The previous study also confirmed OTS964 resistance in GSC population regrowth following administration [14]. In this study, we traced surviving OTS964-administered GS populations where some OTS964-resistant GS clones survived long-term (see Figure 1). Significant fractions of the long-term surviving OTS964-resistant GS clones grew continuously, leading to the GS populations recovering to and eventually surpassing their initial size. This suggests that long-term sequential and repeated administration of OTS964 alone would not control GSC population recurrence and raises a critical question of how the regrowth of GSC populations following OTS964 administration can be controlled.

**Figure 9:**
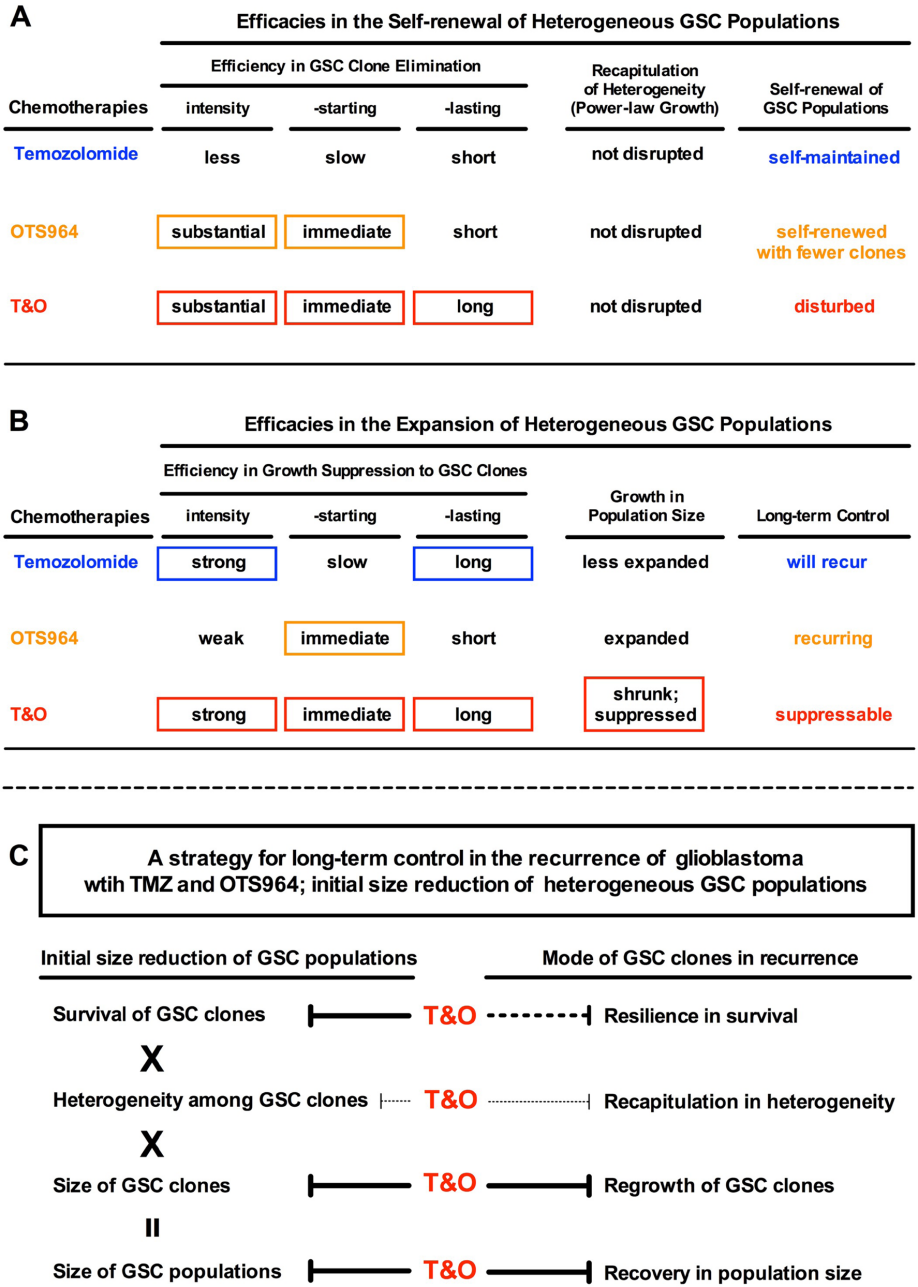
Combined administration of TMZ and OTS964 suppresses the long-term recurrence of GSC populations (**A** and **B**) The tables show the summaries of the efficacy characteristics of TMZ, OTS964 and T&O in heterogeneous GSC population self-renewal and expansion, where T&O disturbs self-renewal and suppresses long-term heterogeneous GSC clone growth. (**C**) A strategy for long-term control of glioblastoma recurrence with TMZ and OTS964 and for the initial size reduction of heterogeneous GSC populations.

Previously and currently we reported that recovered OTS964-administered GS clones did not enhance the probability of survival in following generations even after substantial clone elimination [14]. However, the recovered OTS964-administered GS populations tend to enhance GS clone growth, suggesting it is necessary to suppress GSC clone growth in recurrent GSC populations following OTS964 administration. In this study, we found that TMZ strongly suppressed the growth of GS clones. Moreover, its ability to disturb growth was stable across multiple generations, suggesting TMZ may be able to control the growth of GSC clones in recurring OTS964-administered GSC populations [35]. This strong and stable growth-disturbing property of TMZ contrasts with OTS964, which only exhibits short-term growth disturbance. On the other hand, OTS964 is more effective at eliminating clones than TMZ. Moreover, OTS964 both eliminates clones and disturbs growth immediately following administration, while TMZ only influences growth 2 days after administration [33, 34]. Thus, TMZ and OTS964 are distinct not only how they eliminate clones and disturb growth, but also in their time to expression of efficacy: TMZ is slow-starting and long-lasting while OTS964 starts immediately but is short-lasting [11, 14, 33, 34] (See Figure 9A and 9B). This suggests the combined administration of TMZ and OTS964 may be able to compensate for the complementary clone elimination and growth disturbance properties and time-windows of the two compounds administered separately.

Combined administration of TMZ and OTS964 (T&O) more significantly reduced GS population size compared to TMZ or OTS964 alone. T&O administration efficiently eliminated GS clones and stably disturbed clone growth, suggesting that combined administration successfully leverages the distinct efficacies of both compounds. Moreover, combined administration exhibited immediate and continuous growth disturbance and reduction in long-term clone survival. The sustained clone elimination and growth disturbance observed suggests a therapy is plausible where GSC population size is controlled through long-term administration of combined TMZ and OTS964 (See Figure 9A and 9B) [40, 41].

### Functional heterogeneity and plasticity of glioma stem cell populations in sensitivity and resistance to TMZ and/or OTS964

We previously reported that GSC populations are heterogeneous and plastic in their chemoresistance to OTS964 [4, 14, 22, 28–30]. GSC populations have been resistant to OTS964 in every generation following administration, with GSC clones surviving and consistently growing to recover their initial population size even after substantial clone elimination. This study also showed that OTS964-resistant GS clones survived long-term, recovering from and surpassing their initial population sizes. This suggests that GSC populations are functionally heterogeneous in their sensitivity and resistance to OTS964, that they preserve their heterogeneity and chemoresistance over generations, and that the heterogeneity is recapitulated through growth [11]. We previously reported that this resistance can be eliminated through self-renewal of recurrent GSC populations with intermittent administration of OTS964 that skips generations [14], suggesting that GSCs plastically acquire and subsequently lose resistance to OTS964 in later generations. By taking advantage of this plasticity in chemo-resistance, it may be possible to control the long-term recurrence of glioblastoma via GSC population size control through a combination of lower-dose radiation and intermittent administration of OTS964 that gives sufficient time for glioblastoma resistance to fade. However, it remains unclear how long it takes for resistant GSCs to re-acquire sensitivity to OTS964 in recurrent GSC populations.

On the other hand, GS clones resisted elimination despite having their growth significantly disturbed by TMZ. TMZ-resistant GS clones consistently survived despite continuous suppression of their growth in subsequent generations following TMZ administration. This suggests that GSC clones are heterogeneous in their resistance to TMZ, and that TMZ-resistant GSC populations appear to be less plastic in their growth recovery. However, there were some TMZ-resistant GS clones that grew fast enough to reconstruct GSC populations. Thus, the survival of GSC clones and the presence of normally expanding TMZ-resistant GS clones suggest that TMZ-resistant GSC populations can quickly recover to exceed pre-treatment GSC populations via relatively rare yet highly plastic TMZ-resistant clones.

Combined administration of TMZ and OTS964 significantly and continuously eliminated GS clones with low long-term survival rates, suggesting GSC populations appear heterogeneous in their resistance to T&O. The growth of T&O-resistant GS clones is continuously suppressed, suggesting reduced recovery with combined TMZ and OTS964 administration compared to their independent administration. Among more than 40,000 T&O-administered self-renewing GS clones in this study, only one fast-growing resistant GS clone was detected. The relatively low level of GSC clone survival and the relative rarity of fast-growing resistant GS clones suggests that combined T&O administration would contribute to longer-term suppression of GS population size. Thus, combined administration of TMZ and OTS964 could efficiently make GSC clones less plastic and continuously suppress GSC population re-growth.

### Robustness of glioma stem cell populations during chemotherapy through preserving a power-law coded heterogeneity

We have discovered power-law growth in heterogeneous GSC populations, representing a plastic reproducibility in the growth of GSCs in both space and time [11, 14, 43]. Thus, power-law coded heterogeneous GSC populations are robust. We then proposed that disruption of this power-law growth, where the scale-free power-law based network is disrupted, may lead to collapse of entire GSC populations. In this study many long-term surviving OTS964-resistant GS clones were fast growing, indicating these clones may have been selected for and enriched through OTS964 exposure. However, expanded GS clone-derived cells at every time point reproduced a power-law growth, suggesting that fast-growing GSC clones preserve the power-law growth property of untreated GSC populations. This again suggests that OTS964 alone is insufficient to disrupt power-law growth in GSC populations [14].

This study showed that both TMZ alone and OTS964 alone shrank the self-renewed and expanded GS populations over different time scales, although the OTS964-administered populations quickly recovered to their original size. Combined administration of TMZ and OTS964 quickly shrank and suppressed the regrowth of the reduced GS populations. However, the frequency distributions of all three, TMZ alone, OTS964 alone, and T&O exhibited power-law growth, with regression lines surprisingly tightly associated and of similar shape following administration. This suggests the power-law growth in the self-renewed and expanding GSC populations was not disrupted, although the compounds significantly shrank the population sizes (see Figure 9). Maintenance of power-law growth following drug administration suggests that the heterogeneous growth properties stabilize in TMZ-, OTS964- and T&O-resistant GSC populations. Thus, the GSC populations preserve power-law growth during resistance acquisition to TMZ and/or OTS964, and so the populations remain robust despite responding to the chemotherapies (see Figure 9) [43, 44].

### A therapeutic strategy for treating glioblastoma using combined TMZ and OTS964

Glioblastomas resist chemoradiotherapy then recur to become fatal space-occupying lesions, meaning concerns about resistance to chemoradiotherapies and tumor size need to be resolved [11, 14, 20]. GSCs are believed to be responsible for the recurrence of tumors as resistant cell populations following chemoradiotherapy, suggesting that the issues of glioblastoma chemoradioresistance and population size should be addressed. The immediate and sustained shrinkage of the self-renewed and expanding GSC populations through combined administration of TMZ and OTS964 suggests a combination therapy may be advantageous for long-term GSC population size control (see Figure 9), and so may represent a novel paradigm for glioblastoma chemotherapy. There would be three major steps in this paradigm to control the long-term regrowth of GSC populations: first, initial chemotherapy administration should substantially shrink the GSC population size early and as quickly as possible; second, GSC clone regrowth should be controlled, with slower growth rates better; third, the GSC populations should not reproduce another generation, leading to a continuous and “hopefully irreversible” shrinkage of the plastically reproducing GSC populations through self-renewal interference and restriction (See Figure 9). This study revealed that shrinkage was best achieved via synergistic GSC clone elimination and immediate and sustainable growth suppression with combined T&O administration compared to TMZ alone or OTS964 alone. Moreover, the growth of T&O-resistant GSC populations was continuously suppressed, suggesting that T&O-resistant GSC populations regrow more slowly from fewer and smaller GSC clones than TMZ- or OTS964-resistant clones, which are more populous and larger. Thus, combined TMZ and OTS964 administration appears preferable to TMZ alone or OTS964 alone, arising from a synergy of their different chemotherapeutic efficacies [30, 37–41, 45–49] (See Figure 9).

Even though the T&O-resistant populations were smaller and grew more slowly, about 1/3 of the GSC clones survived one week following administration into self-renewed and expanding GSC populations. These remaining GSC clones may grow continuously, suggesting that T&O-resistant GSC populations may be able to recover to the size of pre-administration populations in a matter of weeks following initial drug administration. Thus, the one-time administration of T&O would likely be insufficient for long-term regrowth control of some GSC populations, suggesting another paradigm or therapy for controlling of regrowth and reproductive self-renewal remains necessary.

This study revealed that sequential administration of T&O does not substantially eliminate GSC clones, suggesting that sequential administration of T&O would not repeatedly shrink resilient GSC populations via repeated clone elimination, while there is possibly some mild suppression of GSC clone regrowth. We previously proposed a therapy where intermittent administration of OTS964 with low-dose radiation may repeatedly eliminate GSC clones via re-acquisition of sensitivity to OTS964 [14]. This raises another issue: How to control the regrowth of recurrent GSC populations while waiting for GSCs to reacquire OTS964 sensitivity. This study suggests that T&O is preferable for initial size reduction of GSC populations and sustainable control of GSC clone regrowth is possible using TMZ. These suggest chemotherapy paradigms in which TMZ may be administered in the intervals between administrations of OTS964 or T&O, with TMZ alone potentially able to suppress re-growth of OTS964- or T&O-resistant GSC clones during these intervals. Intermittent administrations of OTS964 in combination with TMZ may repeatedly reduce GSC clone size, leading to repeated shrinkage of GSC populations via elimination and sustainable suppression of OTS964- or T&O-resistant GSC clone self-renewal across generations. These issues should be addressed in future studies.

Another important issue is the timing of the radiation therapies [19, 50]. If intermittent administration with a combination of TMZ and OTS964 may repeatedly shrink GSC population size, the timing of the radiation therapy may be a key issue for long-term control of glioblastoma recurrence. If radiation is administered concomitantly with the first administration of OTS964 or T&O, the most substantial reduction in tumor size would likely be seen in the initial phase of treatment. However, the radiation may reduce GSC plasticity, leading to a longer time being required for reacquisition of drug sensitivity, with the risk that the surviving populations could recover and expand during this relatively longer time interval. On the other hand, intermittent administration with TMZ and OTS964 combined may be advantageous for repeated tumor size reduction, which it may be possible to combine with intermittent “lower-dose radiation”. The potential of combined chemoradiotherapy is to be addressed in future research.

The methodological advantage of the approach outlined here is that the heterogeneity of GSC growth properties and sensitivity/resistance to chemotherapy can be quantitatively defined, which offers advantages in establishing a size-control therapy for glioblastoma. Using this method, we demonstrated that a combined TMZ and OTS964 therapy represent a novel therapeutic paradigm for potential long-term control of glioblastoma recurrence via repeated and sustained shrinkage of power-law coded heterogeneous GSC populations [49].

## MATERIALS AND METHODS

### Clonal assay and repopulation experiments

The cell lines U87 (RCB419, RIKEN BRC, Tsukuba, Japan; HTB-14, ATCC) and U251 (RCB0461, RIKEN BRC) were used for project continuity [32]. The procedures employed were described in previous studies [11, 14, 27, 35, 51–53]. Single- or fewer-cell clones were considered “GS clones” in this study [11, 14]. The number of clones and number of cells per clone were quantified at days 7, 16, 23, 30 and 37 for U87-, and at days 7, 14, 20, 27, 33 and 54 for U251-GS clones. GS-containing populations were passaged five to six times. Cells derived from each passage were subjected to clonal assays. As previously described, the first and the second generations of U251-derived GS populations were excluded from the experiments because they are slow-growing. Each series of repopulation experiments were repeated at least twice.

TMZ (LKT laboratories, Inc., St Paul, MN, USA) and OTS964 (kindly provided by OncoTherapy Science, Inc., Kawasaki, Japan) were dissolved in DMSO. We first prepared 1000x various concentrations of OTS964 then administered them to culture media in which the final concentration of DMSO was 0.1%. The control also contained 0.1% DMSO.

### Assay for “clone-eliminating” and “growth-disturbing” TMZ and/or OTS964 efficacies with GS clones in self-renewing heterogeneous GS populations

25 μM of TMZ, 300 nM of OTS964 were used for the single compound and combination (T&O) administrations in the assaying culture. The number of cells in each clone was quantified in both the presence and absence of TMZ and/or OTS964. We determined the concentrations to use consistent with previous studies [11, 14]. 25 μM of TMZ and/or 300 nM of OTS964 significantly reduced the number of both U87- and U251-derived GS clones to 1/3 to 1/5, enabling recollection of sufficient surviving GS clones for the following “recovery/resistant” experiments. We subsequently use the “TMZ-, OTS964- and T&O-administered” GS populations for the resistant GS populations after exposure to TMZ and/or OTS964. The “clone-eliminating” and “growth-disturbing” effects of TMZ and/or OTS964 on GS clones in tumor neurospheres were identified as described previously [14]. This series of experiments was repeated at least twice (from 1st to 6th generations for U87; 3rd to 8th for U251 in each series).

### Assay for “recovery” and “resistance” of GS clones after administration of TMZ and/or OTS964

U87-derived GS clones were treated with TMZ, OTS964 or T&O (@ (n-1)) then the surviving “TMZ-, OTS964- and T&O-administered” GS clones were passaged and dissociated for following generations (@ n for assay). The dissociated GS clones were allowed to grow in the absence of the chemotherapy compounds. In other words, the GS clones were “released” (referred to as “TMZ-, OTS964-, and T&O-released” GS clones) [11, 14]. Thus, we could address whether the surviving “TMZ-, OTS964-, and T&O-administered” GS populations recovered their population size [11, 14]. By contrast, the surviving GS clones were allowed to grow in the presence of the drugs again: namely, the GS clones were sequentially designated as “re-administered” TMZ and/or OTS964 (referred to as “sequentially-administered” GS clones) for whether the surviving “TMZ-, OTS964-, and T&O-sequentially-administered” GS clones exhibited resistance to sequential exposure of the drugs during growth recovery [14]. This series of experiments was repeated at least twice.

### Assay for size reducing effects following administration of TMZ and/or OTS964 on self-renewed and expanded GS populations

U87- and U251-GS clones were grown for 4 days (day 4 experiments), and U251-GS clones for 14 days (day 14 experiments) to become self-renewed and expanded GS populations prior to TMZ and/or OTS964 administration. The drug-administered GS populations were assayed at 2 and 6 days following administration (days 6 and 10 for the day 4 experiments), or at 3 and 6 days following administration (days 17 and 20 for the day 14 experiments).

### Graphs and statistical analyses

All graphing and regression analyses used GraphPad Prism version 5.0b for Mac OS X (GraphPad Software, Inc., San Diego California USA, https://www.graphpad.com.).

## SUPPLEMENTARY MATERIALS FIGURES


