# Supplementary material for: The combined efficacy of OTS964 and temozolomide for reducing the size of power-law coded heterogeneous glioma stem cell populations
Source: Oncotarget. 2019 Mar 22;10(24):2397–415. doi: 10.18632/oncotarget.26800 (PMC6481323; doi:10.18632/oncotarget.26800)
Supplement: Supplementary file 1 [file oncotarget-10-2397-s001.pdf]

The combined efficacy of OTS964 and temozolomide for reducing the size of power-law coded heterogeneous glioma stem cell populations

SUPPLEMENTARY MATERIALS

Temozolomide alone and administration of temozolomide and OTS964 in combination efficiently control the recurrent growth of heterogeneous GS population size via stable suppression of GS clone growth

We previously reported that the recurrent growth of "surviving TMZ-administered GS clones (TMZ-survived GS clones)" recapitulated a power-law growth. We also reported that OTS964-survived GS clones recovered GS population size in following generations with reduced survivability and increased GS clone recovery. We first asked whether TMZ-survived GS clones GS clones recovered their populations in following generations. We found that surviving TMZ-administered U87-GS clones did not recover GS population size to those of the control populations (Supplementary Figures 5A, 5B and 6A; see blue and black circles). TMZ-survived U87-GS clones exhibited less survivability and slower growth tendencies (Supplementary Figure 5D, 5E, 5G 5H, 6B and 6C). On the other hand, we found that OTS964-survived U87-GS clones recovered their population size, while maintaining GS clone survival and growth tendencies (Supplementary Figures 5A, 5B, 5D, 5E, 5G, 5H, 6; see orange and black circles; Sugimori *et al* 2018). We then asked whether/how T&O-survived U87-GS populations recovered GS population size in following generations. T&O-survived GS clones did not recover their population size as robustly as TMZ alone. Both the TMZ only and T&O administered populations maintained growth suppression in surviving U87-GS clones, while about half the GS clones survived compared to control populations in following generations (Supplementary Figures 5A, 5B, 5D, 5E, 5G, 5H and 6; see blue, red and black circles). This suggests that GS populations may be controlled long-term through treatment with TMZ via suppressing GS clone growth across generations, even if a considerable amount of surviving TMZ- or T&O-administered GS clones remain in each following generation.

We previously reported that OTS964-survived GS populations were resistant to sequential administration of OTS964 during recovery and recurrent growth, especially in clone-elimination (Supplementary Figures 5B, 5C, 5E, 5F, 5H, 5I and 6; see orange circles). We then asked whether TMZ- or T&O-survived U87-GS clones resisted sequential administration as well as OTS964-survived recovering and recurrent GS populations. Both TMZ- and T&O-survived recovering and recurrent GS clones exhibited similar survival probabilities in the sequential administration and the single administration paradigms (Supplementary Figures 5B, 5C, 5E, 5F, 5H, 5I, 6; see blue and red circles). This suggests that both TMZ- and T&O-survived U87-GS populations were resistant to the drugs in following generations. The data also indicate that addition of TMZ may solve the problem in which OTS964-survived GS clones continuously re-grow to quickly recover and/or surpass their original population size in following generations.

We next asked whether the various GS populations follow a power-law in their growth. Every regression line for frequency distribution of drug-administered, single administration and sequential administration GS populations exhibited power-law growth (Supplementary Figure 7), showing that any U87-GS population treated with TMZ and/or OTS964 follows a power-law growth. Slopes for TMZ and T&O administered GS populations were steeper than those for OTS964-administered populations, suggesting less diversity in growth in TMZ and T&O-administered GS populations. However, coefficient variations for the frequency distributions of TMZ and T&O administered GS populations were considerably higher than for those of OTS964-administered populations, suggesting that even through TMZ and T&O-administered GS populations did not grow faster, their GS populations maintained diversity in their growth. Thus, no paradigm, TMZ, OTS964 or T&O, disrupted diversity in growth or the following power-law growth of GSC populations.

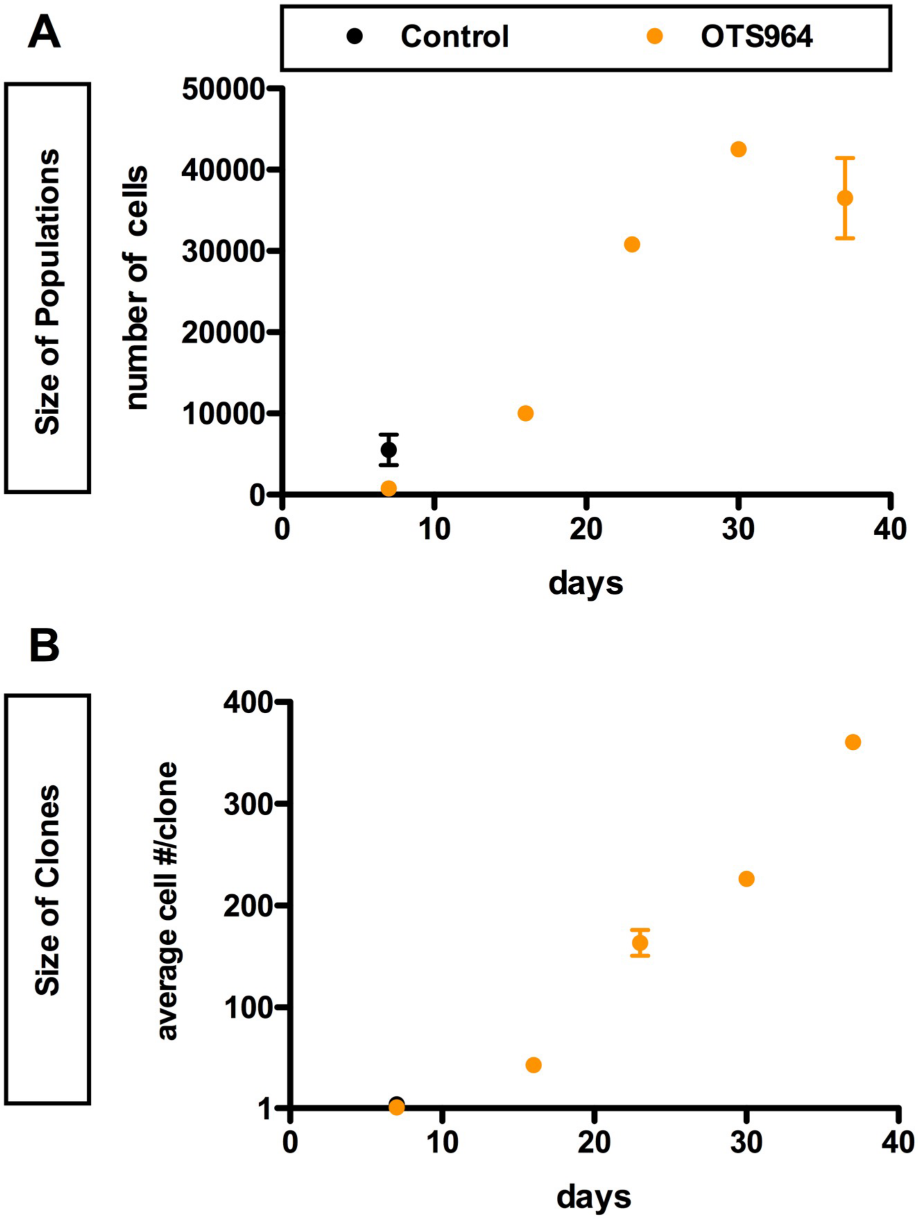

Supplementary Figure 1: Surviving OTS964-adminstered U87-GS clones continuously grow to expand GS population size. (**A**) The graph shows the approximately weekly expansion of U87-GS population size. Data from 16 or more days includes the cell number of very large clones, which could not be precisely quantified. The size of clone populations represents the total cell number from dissociated surviving OTS964-administered GS clones, when we recovered surviving OTS964-administered GS clones. (**B**) The graph shows the approximately weekly growth of U87-GS clones. As described above, we quantified the total cell number of expanded GS populations. Then, the average size of clones was determined by dividing the total population size by the number of clones.

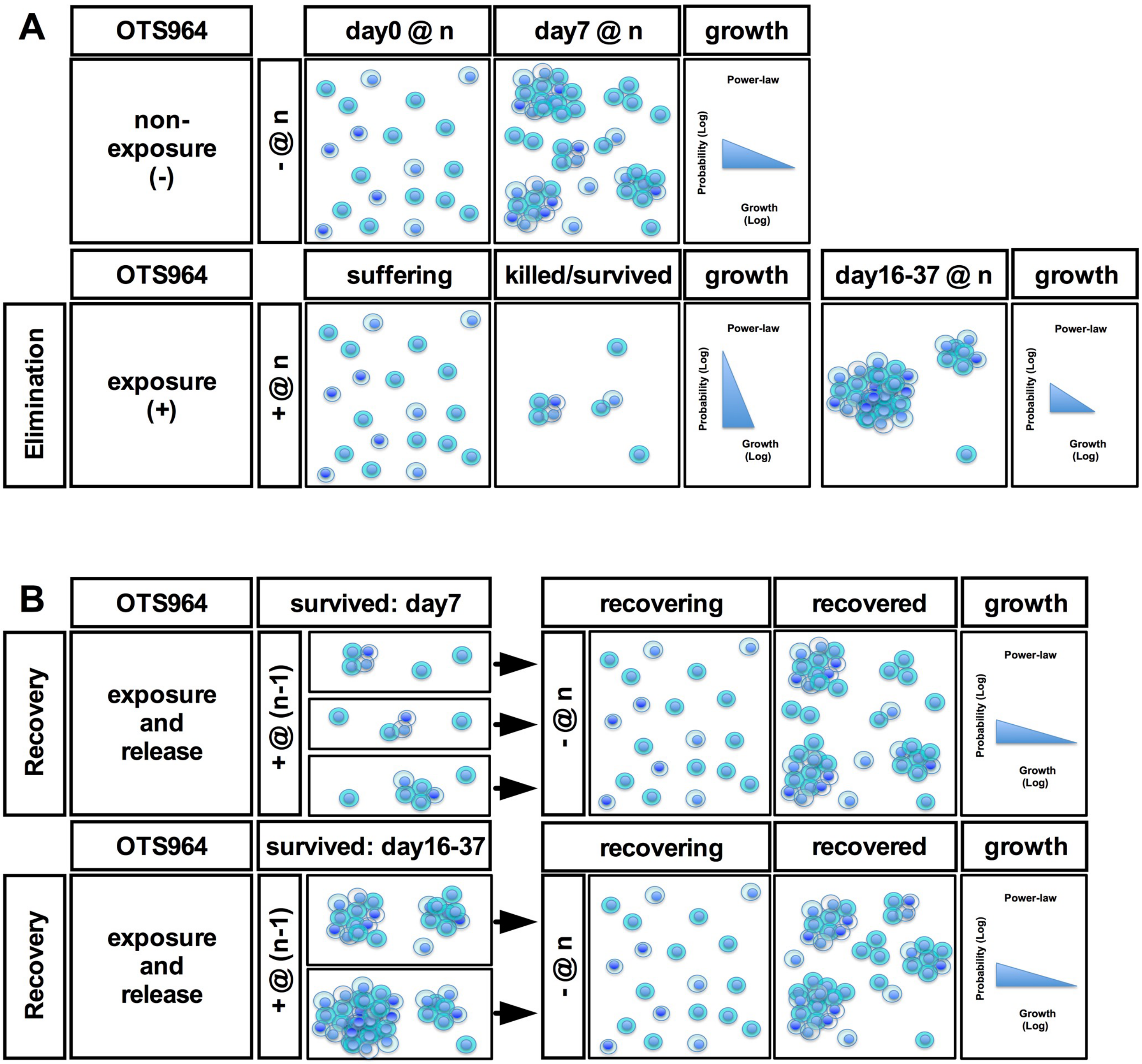

Supplementary Figure 2: OTS964 kills U87-GS clones and disturbs early growth, but is not able to stop later growth of surviving OTS964-administered GS clones in the tumor neurosphere culture. (**A**) U87-GS clones were assayed in the absence/presence of 300 nM of OTS964 (see above for the absence; and below for the presence, respectively) at an assay (@ n for an assay) generation. Following some limited GS clone elimination, surviving OTS964-administered GS clones later grow to become large clones (days 16, 23, 30 and 37). U251-GS clones recapitulated similar phenotypes following elimination and then later clonal expansion (see Figures 1 to 3). (**B**) OTS964-survived U-87GS populations recovered while maintaining a power-law. Schematic diagrams show the recovery-assaying experiments in series. GS populations were exposed to OTS964 (@ (n-1)) for 7 (top) and 16, 23, 30 and 37 days (bottom), then passaged/dissociated to conduct assays for following generations (@ n). OTS964-administered GS populations from "each-day experiments" recovered their size while maintaining a power-law growth.

A

Size of Populations

number of cells		U87 (day 7) TMZ	U87 (day 7) OTS964	U87 (day 7) T&O
Elimination	Control	**	**	**
	TMZ		ns	ns
	OTS964			ns

number of cells		U251 (day 7) TMZ	U251 (day 7) OTS964	U251 (day 7) T&O
Elimination	Control	**	**	**
	TMZ		**	**
	OTS964			ns

number of cells		U251 (day 20) TMZ	U251 (day 20) OTS964	U251 (day 20) T&O
Elimination	Control	**	**	**
	TMZ		ns	*
	OTS964			ns

B

Number of Clones

number of clones		U87 (day 7) TMZ	U87 (day 7) OTS964	U87 (day 7) T&O
Elimination	Control	**	**	**
	TMZ		**	**
	OTS964			ns

number of clones		U251 (day 7) TMZ	U251 (day 7) OTS964	U251 (day 7) T&O
Elimination	Control	**	**	**
	TMZ		**	**
	OTS964			ns

number of clones		U251 (day 20) TMZ	U251 (day 20) OTS964	U251 (day 20) T&O
Elimination	Control	**	**	**
	TMZ		**	**
	OTS964			ns

C

Size of Clones

average cell#/clone		U87 (day 7) TMZ	U87 (day 7) OTS964	U87 (day 7) T&O
Elimination	Control	**	**	**
	TMZ		ns	ns
	OTS964			*

average cell#/clone		U251 (day 7) TMZ	U251 (day 7) OTS964	U251 (day 7) T&O
Elimination	Control	**	**	**
	TMZ		**	**
	OTS964			ns

average cell#/clone		U251 (day 20) TMZ	U251 (day 20) OTS964	U251 (day 20) T&O
Elimination	Control	**	*	**
	TMZ		ns	ns
	OTS964			ns

Supplementary Figure 3: Statistical analyses for Figure 3. **(A–C)** The tables show the results of One-way ANOVA analysis where the data from four different administration paradigms were compared for differences in population size (A), the number of clones (B) and clone size (C). The data came from day 7 for U87-GS clones (as shown in the Figures 3A, 3D and 3G), and days 7 (Figure 3B, 3E and 3H) and 20 (Figure 3C, 3F and 3I) for U251-GS clones. The asterisks indicate the results of comparison using Bonferroni's Multiple Comparison test: $^{*}P < 0.05$; $^{**}P < 0.01$; ns: not significant.

A

U87

number of clones		day 16		day 23			day 30			day 37		
		OTS964	T&O	TMZ	OTS964	T&O	TMZ	OTS964	T&O	TMZ	OTS964	T&O
day 16	TMZ	ns	**	ns	**	**	**	**	**	**	**	**
	OTS964		**	ns	ns	**	ns	ns	**	**	*	**
	T&O			**	*	ns	*	ns	ns	ns	ns	ns
day 23	TMZ				ns	**	ns	*	**	ns	ns	**
	OTS964					**	ns	ns	**	ns	ns	**
	T&O						**	ns	ns	*	ns	ns
day 30	TMZ							ns	**	ns	ns	**
	OTS964								*	ns	ns	*
	T&O									**	ns	ns
day 37	TMZ										ns	**
	OTS964											ns

B

U251

number of clones		day 20		day 27			day 33			day 54		
		OTS964	T&O	TMZ	OTS964	T&O	TMZ	OTS964	T&O	TMZ	OTS964	T&O
day 20	TMZ	**	**	ns	**	**	ns	**	**	**	**	**
	OTS964		ns	**	ns	ns	**	ns	ns	**	ns	ns
	T&O			**	ns	ns	**	ns	ns	**	ns	ns
day 27	TMZ				**	**	ns	**	**	**	**	**
	OTS964					ns	**	ns	ns	**	ns	ns
	T&O						**	ns	ns	**	ns	ns
day 33	TMZ							**	**	ns	**	**
	OTS964								ns	**	ns	ns
	T&O									**	ns	ns
day 54	TMZ										**	**
	OTS964											ns

Supplementary Figure 4: Statistical analyses for the graphs in Figure 4A–4H. (**A** and **B**) The tables show the results of One-way ANOVA analysis where 12 different data points (3 different administration paradigms with 4 assayed days for each paradigm) were compared for differences in the number of clones. The data are derived from U87- (A) and U251-GS clones (B), respectively. The letters shown in black represent comparisons with one parameter of difference (ex. TMZ at "day 16" vs TMZ at "day 23"; "TMZ" vs "OTS964" at day 16), while the gray letters represent comparisons between data with more than one parameters of difference (ex. "TMZ" at "day 16" vs "OTS964" at "day 23"). $^{*}P < 0.05$; $^{**}P < 0.01$; ns: not significant using Bonferroni's Multiple Comparison Test.

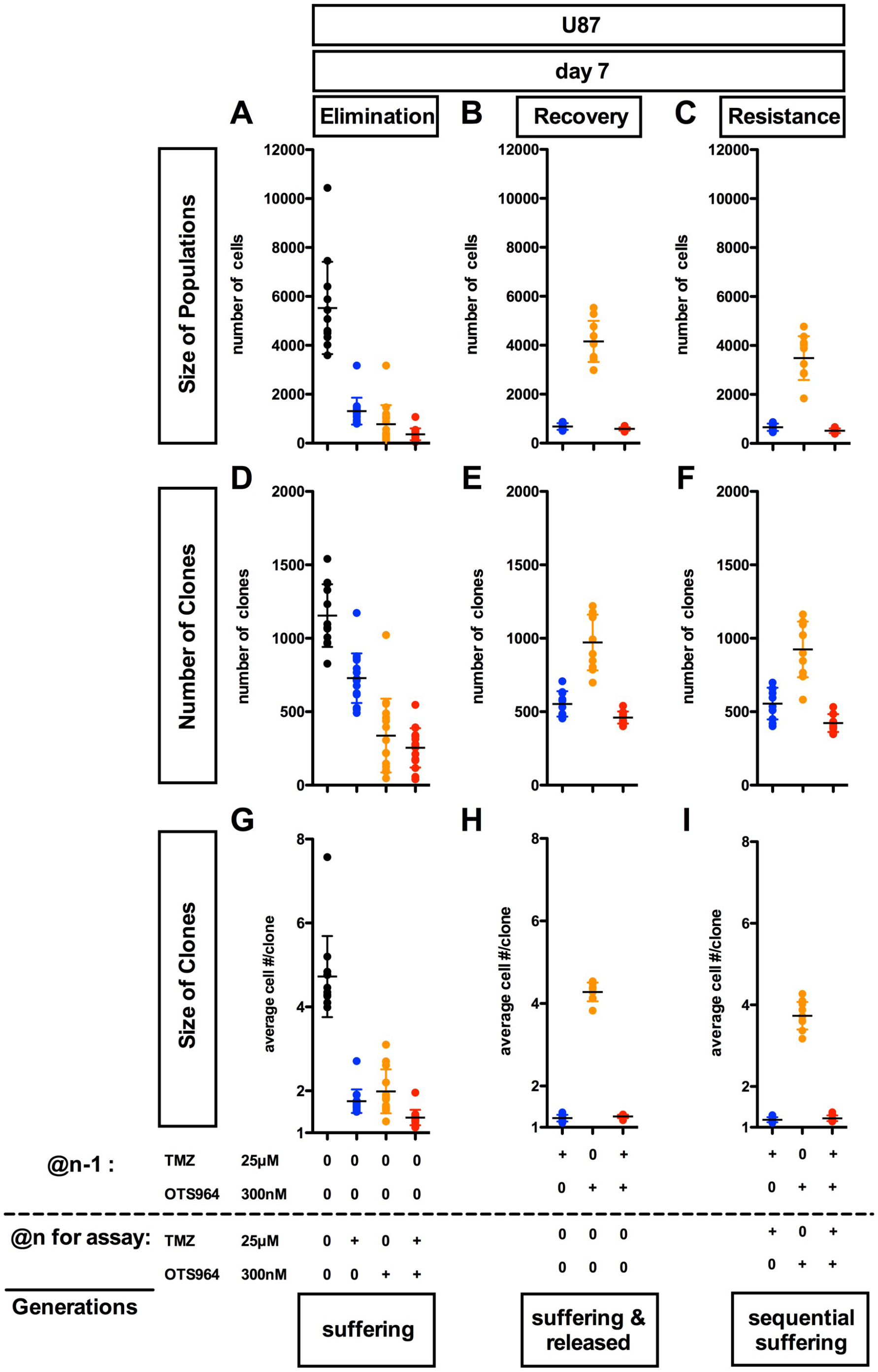

Supplementary Figure 5: TMZ and T&O suppressed the expansion of U87-GS populations across generations via continuous suppression in GS clone growth; The surviving TMZ-, OTS964- and T&O-administered GS populations resisted sequential administration during growth recovery. (**A–I**) The graphs show the size of U87-GS populations (A–C), the number of GS clones (D–F) and the size of GS clones (G–I), respectively. (A, D and G) 300 nM of OTS964 reduced GS populations via elimination of GS clones and suppression of GS clone growth (orange circles), while 25 µM of TMZ also reduced the size of the GS populations via significant suppression of GS clone growth and survival (blue circles). However, TMZ's clone-eliminating effect was smaller than that of OTS964 (A, D and G; A, D and G are the same graphs in Figures 3A, 3D and 3G for comparison to the other graphs in Supplementary Figure 5; see also Sugimori et al, 2015; 2018). T&O in combination was stronger than single administration of OTS964 or TMZ for eliminating clones and disturbing their growth (red circles). (B, E and H) TMZ- and T&O-survived GS population expansion was suppressed during GS clone growth recovery. Surviving TMZ-, OTS964- and T&O-administered GS populations were passaged and released from the drugs to recover their population size: "suffering & released". The TMZ- and T&O-survived GS populations did not expand well via strong suppression of the growth of clones, while the GS clones survived (blue and red circles). On the other hand, OTS964-survived GS clones consistently survived and grew to recover their population size as described previously (orange circles; see also Sugimori *et al*, 2018). (C, F and I) The TMZ-, OTS964- and T&O-survived GS populations resisted the sequential administration paradigms during recovery. The TMZ-, OTS964- and T&O-survived GS populations maintained GS clones survival and growth in the sequential administration paradigms and the single administration paradigms.

A — Size of Populations

number of cells		Elimination			Recovery			Resistance		
		TMZ	OTS964	T&O	TMZ	OTS964	T&O	TMZ	OTS964	T&O
	Control	**	**	**	**	**	**	**	**	**
Elimination	TMZ		ns	*	ns	**	ns	ns	**	ns
	OTS964			ns	ns	**	ns	ns	**	ns
	T&O				ns	**	ns	ns	**	ns
Recovery	TMZ					**	ns	ns	**	ns
	OTS964						**	**	ns	**
	T&O							ns	**	ns
Resistance	TMZ								**	ns
	OTS964									**

B — Number of Clones

number of clones		Elimination			Recovery			Resistance		
		TMZ	OTS964	T&O	TMZ	OTS964	T&O	TMZ	OTS964	T&O
	Control	**	**	**	**	ns	**	**	ns	**
Elimination	TMZ		**	**	ns	*	**	ns	ns	**
	OTS964			ns	ns	**	ns	ns	**	ns
	T&O				**	**	ns	**	**	ns
Recovery	TMZ					**	ns	ns	**	ns
	OTS964						**	**	ns	**
	T&O							ns	**	ns
Resistance	TMZ								**	ns
	OTS964									**

C — Size of Clones

average cell #/clone		Elimination			Recovery			Resistance		
		TMZ	OTS964	T&O	TMZ	OTS964	T&O	TMZ	OTS964	T&O
	Control	**	**	**	**	ns	**	**	**	**
Elimination	TMZ		ns	ns	ns	**	ns	*	**	ns
	OTS964			**	**	**	**	**	**	**
	T&O				ns	**	ns	ns	**	ns
Recovery	TMZ					**	ns	ns	**	ns
	OTS964						**	**	ns	**
	T&O							ns	**	ns
Resistance	TMZ								**	ns
	OTS964									**

Supplementary Figure 6: Statistical analyses for Supplementary Figure 5. (**A–C**) The tables show the results of One-way ANOVA analysis where data from 10 different administration paradigms were compared to examine differences in population size (A), clone number (B) and clone size (C). The data is from U87-GS clones at day 7 as shown in Supplementary Figure 5. The letters in black represent comparisons with one parameter of difference (ex. TMZ at "Elimination" vs TMZ at "Recovery"; "TMZ" vs "OTS964" at Elimination). The gray letters represent comparisons with more than one parameters of difference (ex. "Control" at "Elimination" vs "TMZ" at "Resistance"; "TMZ" at "Elimination" vs "OTS964" at "Recovery"). $^{*}P < 0.05$; $^{**}P < 0.01$.; ns: not significant using Bonferroni's Multiple Comparison Test.

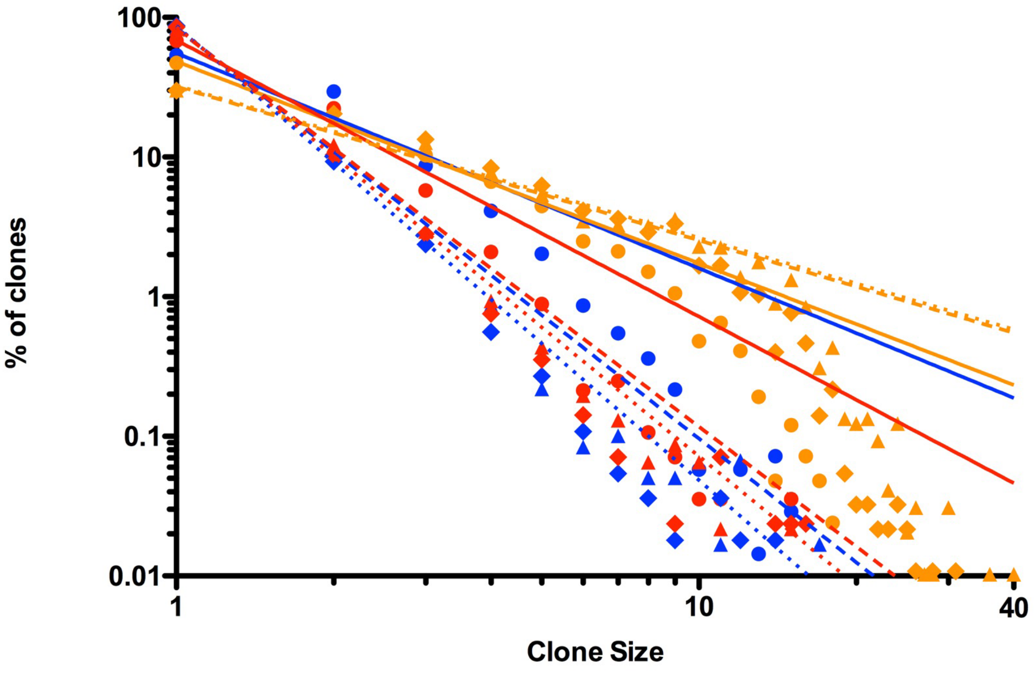

administration paradigm	n-1 → n (assay)	TMZ	OTS964	T&O
suffering	0 → +	—●—	—●—	—●—
suffering & released	+ → 0	-▲-	-▲-	-▲-
sequential suffering	+ → +	-◆-	-◆-	-◆-

administration paradigm	R^2			CV (%)		
	TMZ	OTS964	T&O	TMZ	OTS964	T&O
suffering	0.96	0.99	0.99	383	330	449
suffering & released	0.99	0.96	0.99	536	231	528
sequential suffering	1.00	0.95	0.99	553	240	542

Supplementary Figure 7: Recapitulation of diversity and power-law in the growth in the drug-suffering, -recovered and -resistant GS populations. The graph shows double logarithmic plots of the clone size (number of cells in each clone) and the frequency of the U87 derived from GS populations for three different drug-administration paradigms: "suffering" where drugs were administered at assay-generation (n) (shown in triangles); "suffering and released" at the previous generation (n-1) before an assay (triangles); "sequential suffering: seq-suffering" at sequential generations before and at an assay (diamonds). The GS populations of "suffering", "suffering and released" and "sequential suffering: seq-suffering" paradigms show "drug-suffering", "-survived & recovered" and "-resistant" phenotypes at day 7. The regression lines for drug-suffering, - survived & recovered and -resistant GS populations are shown in solid, dashed and dotted lines, respectively. Administration of 25 μM of TMZ, 300 nM of OTS964 and combination of TMZ and OTS964 are shown in blue, orange and red labels/lines, respectively. The every drug-suffering, -recovered, -resistant GS population follows a power-law. Coefficient determinations ($R^{2)}$ for the regression lines are shown (left part); Coefficient variations (CV) for the frequency distributions are shown (right part) in the table below.

U87

A

Size of Populations

number of cells		day 6 (post_day 2)				day 10 (post_day 6)			
		Control	TMZ	OTS964	T&O	Control	TMZ	OTS964	T&O
day 4	Control	*	ns	ns	**	**	**	ns	**
day 6 (post_day 2)	Control		ns	**	**	**	**	ns	**
	TMZ			ns	**	**	**	ns	**
	OTS964				ns	**	ns	**	*
	T&O					**	ns	**	ns
day 10 (post_day 6)	Control						**	**	**
	TMZ							**	ns
	OTS964								**

B

Number of Clones

number of clones		day 6 (post_day 2)				day 10 (post_day 6)			
		Control	TMZ	OTS964	T&O	Control	TMZ	OTS964	T&O
day 4	Control	ns	**	**	**	**	**	**	**
day 6 (post_day 2)	Control		ns	**	**	**	**	**	**
	TMZ			ns	**	ns	**	**	**
	OTS964				ns	ns	**	**	**
	T&O					ns	ns	ns	**
day 10 (post_day 6)	Control						ns	ns	**
	TMZ							ns	ns
	OTS964								ns

C

Size of Clones

average cell #/clone		day 6 (post_day 2)				day 10 (post_day 6)			
		Control	TMZ	OTS964	T&O	Control	TMZ	OTS964	T&O
day 4	Control	**	**	**	*	**	**	**	**
day 6 (post_day 2)	Control		ns	ns	*	**	ns	**	ns
	TMZ			ns	ns	**	ns	**	ns
	OTS964				ns	**	*	**	ns
	T&O					**	**	**	**
day 10 (post_day 6)	Control						**	**	**
	TMZ							**	ns
	OTS964								**

Supplementary Figure 8: Statistical analyses for Figures 5B–5D. (**A–C**) The tables show the results of One-way ANOVA analysis of 9 different data groups, in which 4 different administration paradigms examined at 3 different assaying days were compared for differences in population size (A), clone number (B) and clone size (C). The data in Figures 5B–5D is from U87-GS clones at day 4, representing the populations' pre-administration status; at days 6 and 10, representing populations for 2 and 6 days following administration (post_day 2 and post_day 6), respectively. The letters in black represent comparisons with one parameter of difference {ex. Control at "day 4" vs TMZ at "day 6 (post_day 2)"; "TMZ" vs "OTS964" at day 6 (post_day 2) }, while the gray letters represent comparisons between the data with more than one parameters of difference {ex. "TMZ" at "day 6 (post_day 2)" vs "OTS964" at "day 10 (post_day 6)" }. $^{*}P < 0.05$; $^{**}P < 0.01$; ns: not significant using Bonferroni's Multiple Comparison Test.

Size of Populations

A — U251; day 4 experiments

number of cells		day 6 (post_day 2) Control	day 6 (post_day 2) TMZ	day 6 (post_day 2) OTS 964	day 6 (post_day 2) T&O	day 10 (post_day 6) Control	day 10 (post_day 6) TMZ	day 10 (post_day 6) OTS 964	day 10 (post_day 6) T&O	day 38 (post_day 34) TMZ	day 38 (post_day 34) OTS 964	day 38 (post_day 34) T&O	day 46 (post_day 42) TMZ	day 46 (post_day 42) OTS 964	day 46 (post_day 42) T&O
day 4	Control	ns	ns	ns	ns	*	ns	ns	**	ns	ns	**	ns	**	**
day 6 (post_day 2)	Control		ns	ns	**	ns	ns	**	**	ns	ns	**	ns	ns	**
	TMZ			ns	ns	ns	ns	*	**	ns	ns	**	ns	ns	**
	OTS964				ns	**	ns	ns	ns	ns	*	ns	ns	**	ns
	T&O					**	ns	ns	ns	ns	**	ns	ns	**	ns
day 10 (post_day 6)	Control						ns	**	**	ns	ns	**	**	ns	**
	TMZ							ns	**	ns	ns	*	ns	ns	**
	OTS964								ns	ns	**	ns	ns	**	ns
	T&O									ns	**	ns	ns	**	ns
day 38 (post_day 34)	TMZ										ns	ns	ns	*	ns
	OTS964											**	*	ns	**
	T&O												ns	**	ns
day 46 (post_day 42)	TMZ													**	ns
	OTS964														**

B — U251; day 14 experiments

number of cells		day 17 (post_day 3) Control	day 17 (post_day 3) TMZ	day 17 (post_day 3) OTS 964	day 17 (post_day 3) T&O	day 20 (post_day 6) Control	day 20 (post_day 6) TMZ	day 20 (post_day 6) OTS 964	day 20 (post_day 6) T&O	day 38 (post_day 24) TMZ	day 38 (post_day 24) OTS 964	day 38 (post_day 24) T&O	day 46 (post_day 32) TMZ	day 46 (post_day 32) OTS 964	day 46 (post_day 32) T&O
day 14	Control	ns	ns	ns	**	*	ns	**	**	ns	**	**	ns	**	**
day 17 (post_day 3)	Control		ns	ns	*	ns	ns	**	**	ns	**	**	ns	**	**
	TMZ			ns	**	ns	ns	**	**	ns	**	**	*	**	**
	OTS964				ns	*	ns	*	**	ns	**	**	ns	**	**
	T&O					**	**	ns	ns	ns	ns	**	ns	ns	**
day 20 (post_day 6)	Control						ns	**	**	ns	**	**	**	**	**
	TMZ							**	**	ns	**	**	**	**	**
	OTS964								ns	*	ns	*	ns	ns	**
	T&O									**	ns	ns	**	ns	ns
day 38 (post_day 24)	TMZ										**	**	ns	**	**
	OTS964											ns	**	ns	ns
	T&O												**	ns	ns
day 46 (post_day 32)	TMZ													**	**
	OTS964														ns

Supplementary Figure 9: Statistical analyses for Figure 6A. (**A** and **B**) The tables show the results of One-way ANOVA analysis of 15 different data groups, in which 4 different administration paradigms were examined on 5 different assaying days to compared differences in U251-derived GS population size for day 4 experiments (A) and for day 14 experiments (B). (A) The data are from the day 4 experiments: day 4 represents the pre-administration populations; days 6, 10, 38 and 46, represent the populations 2, 6, 34 and 42 days following administration (post_day 2, post_day 6, post_day 34 and post_day 42), respectively. (B) The data are from the day 14 experiments: day 14 represents the pre-administration populations; days 17, 20, 38 and 46 represent the populations 3, 6, 24 and 32 days following administration (post_day 3, post_day 6, post_day 24 and post_day 32), respectively. The letters in black represent comparisons with one parameter of difference (ex. Control at "day 4" vs TMZ at "day 6 (post_day 2)"; "TMZ" vs "OTS964" at day 6 (post_day 2)), while the gray letters represent comparisons with more than one parameter of difference (ex. "TMZ" at "day 6 (post_day 2)" vs "OTS964" at "day 10 (post_day 6)"). $^{*}P < 0.05$; $^{**}P < 0.01$; ns: not significant using Bonferroni's Multiple Comparison Test.

Number of Clones

A U251; day 4 experiments

number of clones		day 6 (post_day 2) Control	day 6 (post_day 2) TMZ	day 6 (post_day 2) OTS 964	day 6 (post_day 2) T&O	day 10 (post_day 6) Control	day 10 (post_day 6) TMZ	day 10 (post_day 6) OTS 964	day 10 (post_day 6) T&O	day 38 (post_day 34) TMZ	day 38 (post_day 34) OTS 964	day 38 (post_day 34) T&O	day 46 (post_day 42) TMZ	day 46 (post_day 42) OTS 964	day 46 (post_day 42) T&O
day 4	Control	ns	ns	ns	*	ns	ns	**	**	ns	ns	**	ns	**	**
day 6 (post_day 2)	Control		ns	ns	**	ns	ns	**	**	ns	*	**	*	**	**
	TMZ			ns	ns	ns	ns	**	**	ns	ns	**	ns	**	**
	OTS964				ns	ns	ns	ns	*	ns	ns	*	ns	ns	**
	T&O					*	ns	ns	ns	ns	ns	ns	ns	ns	ns
day 10 (post_day 6)	Control						ns	**	**	ns	ns	**	ns	**	**
	TMZ							ns	**	ns	ns	**	ns	ns	**
	OTS964								ns	ns	ns	ns	ns	ns	ns
	T&O									ns	ns	ns	ns	ns	ns
day 38 (post_day 34)	TMZ										ns	ns	ns	ns	ns
	OTS964											ns	ns	ns	ns
	T&O												ns	ns	ns
day 46 (post_day 42)	TMZ													ns	ns
	OTS964														ns

B U251; day 14 experiments

number of clones		day 17 (post_day 3) Control	day 17 (post_day 3) TMZ	day 17 (post_day 3) OTS 964	day 17 (post_day 3) T&O	day 20 (post_day 6) Control	day 20 (post_day 6) TMZ	day 20 (post_day 6) OTS 964	day 20 (post_day 6) T&O	day 38 (post_day 24) TMZ	day 38 (post_day 24) OTS 964	day 38 (post_day 24) T&O	day 46 (post_day 32) TMZ	day 46 (post_day 32) OTS 964	day 46 (post_day 32) T&O
day 14	Control	ns	ns	**	**	ns	ns	**	**	ns	**	**	**	**	**
day 17 (post_day 3)	Control		ns	ns	**	ns	ns	**	**	ns	**	**	ns	**	**
	TMZ			ns	**	ns	ns	**	**	ns	**	**	ns	**	**
	OTS964				ns	**	*	*	**	ns	**	**	ns	**	**
	T&O					**	**	ns	*	*	**	**	ns	**	**
day 20 (post_day 6)	Control						ns	**	**	*	**	**	**	**	**
	TMZ							**	**	ns	**	**	**	**	**
	OTS964								ns	**	**	**	*	**	**
	T&O									**	ns	*	**	ns	**
day 38 (post_day 24)	TMZ										**	**	ns	**	**
	OTS964											ns	**	ns	ns
	T&O												**	ns	ns
day 46 (post_day 32)	TMZ													**	**
	OTS964														ns

Supplementary Figure 10: Statistical analyses for Figure 6B. (**A** and **B**) The tables show the results of One-way ANOVA analysis of 15 different data groups, in which 4 different administration paradigms were examined on 5 different assaying days to compared differences in the clone numbers of U251-derived GS populations for the day 4 experiments (A) and for the day 14 experiments (B). (A) The data are from the day 4 experiments: day 4 represents the pre-administration populations; days 6, 10, 38 and 46 representing the populations 2, 6, 34 and 42 days following administration (post_day 2, post_day 6, post_day 34 and post_day 42), respectively. (B) The data are from the day 14 experiments: day 14 represents the pre-administration populations; days 17, 20, 38 and 46 represent the populations 3, 6, 24 and 32 days following administration (post_day 3, post_day 6, post_day 24 and post_day 32), respectively. The letters in black represent comparisons with one parameter of difference (ex. Control at "day 4" vs TMZ at "day 6 (post_day 2)"; "TMZ" vs "OTS964" at day 6 (post_day 2)), while the gray letters represent comparisons with more than one parameter of difference (ex. "TMZ" at "day 6 (post_day 2)" vs "OTS964" at "day 10 (post_day 6)"). $^{*}P < 0.05$; $^{**}P < 0.01$; ns: not significant using Bonferroni's Multiple Comparison Test.

Size of Clones

A — U251; day 4 experiments

average cell#/ clone		day 6 (post_day 2)				day 10 (post_day 6)				day 38 (post_day 34)			day 46 (post_day 42)		
		Control	TMZ	OTS 964	T&O	Control	TMZ	OTS 964	T&O	TMZ	OTS 964	T&O	TMZ	OTS 964	T&O
day 4	Control	ns	ns	ns	ns	**	ns	ns	ns	ns	**	ns	ns	**	ns
day 6 (post_day 2)	Control		ns	ns	ns	**	ns	ns	**	ns	**	ns	ns	**	**
	TMZ			ns	ns	*	ns	ns	*	ns	**	ns	ns	**	*
	OTS964				ns	**	ns	ns	ns	ns	**	ns	ns	**	ns
	T&O					**	ns	ns	ns	ns	**	ns	ns	**	ns
day 10 (post_day 6)	Control						*	**	**	ns	**	**	ns	**	**
	TMZ							ns	ns	ns	**	ns	ns	**	*
	OTS964								ns	ns	**	ns	ns	**	ns
	T&O									ns	**	ns	ns	**	ns
day 38 (post_day 34)	TMZ										**	ns	ns	**	ns
	OTS964											**	**	**	**
	T&O												ns	**	ns
day 46 (post_day 42)	TMZ													**	*
	OTS964														**

B — U251; day 14 experiments

average cell#/ clone		day 17 (post_day 3)				day 20 (post_day 6)				day 38 (post_day 24)			day 46 (post_day 32)		
		Control	TMZ	OTS 964	T&O	Control	TMZ	OTS 964	T&O	TMZ	OTS 964	T&O	TMZ	OTS 964	T&O
day 14	Control	ns	ns	ns	ns	ns	ns	ns	ns	ns	ns	ns	ns	**	ns
day 17 (post_day 3)	Control		ns	ns	ns	ns	ns	ns	ns	ns	ns	ns	ns	ns	ns
	TMZ			ns	ns	ns	ns	ns	ns	ns	ns	*	ns	ns	**
	OTS964				ns	ns	ns	ns	ns	ns	ns	ns	ns	ns	ns
	T&O					ns	ns	ns	ns	ns	ns	ns	ns	**	ns
day 20 (post_day 6)	Control						ns	ns	ns	ns	ns	ns	ns	*	*
	TMZ							ns	ns	ns	ns	ns	ns	*	*
	OTS964								ns	ns	ns	ns	ns	**	ns
	T&O									ns	*	ns	ns	**	ns
day 38 (post_day 24)	TMZ										ns	ns	ns	*	ns
	OTS964											**	ns	ns	**
	T&O												ns	**	ns
day 46 (post_day 32)	TMZ													**	ns
	OTS964														**

Supplementary Figure 11: Statistical analyses for Figure 6C. (**A** and **B**) The tables show the results of One-way ANOVA analysis of 15 different data groups, in which 4 different administration paradigms were examined on 5 different assaying days to compared differences in the clone size of U251-derived GS populations for the day 4 experiments (A) and for the day 14 experiments (B). (A) The data are from the day 4 experiments: day 4 represents the pre-administration populations; days 6, 10, 38 and 46 represent the populations 2, 6, 34 and 42 days following administration (post_day 2, post_day 6, post_day 34 and post_day 42), respectively. (B) The data are from the day 14 experiments: day 14 represents the pre-administration populations; days 17, 20, 38 and 46 represent the populations 3, 6, 24 and 32 days following administration (post_day 3, post_day 6, post_day 24 and post_day 32), respectively. The letters in black represent comparisons with one parameter of difference (ex. Control at "day 4" vs TMZ at "day 6 (post_day 2)"; "TMZ" vs "OTS964" at day 6 (post_day 2)), while the gray letters represent comparisons with more than one parameter of difference (ex. "TMZ" at "day 6 (post_day 2)" vs "OTS964" at "day 10 (post_day 6)"). $^{*}P < 0.05$; $^{**}P < 0.01$; ns: not significant using Bonferroni's Multiple Comparison Test.

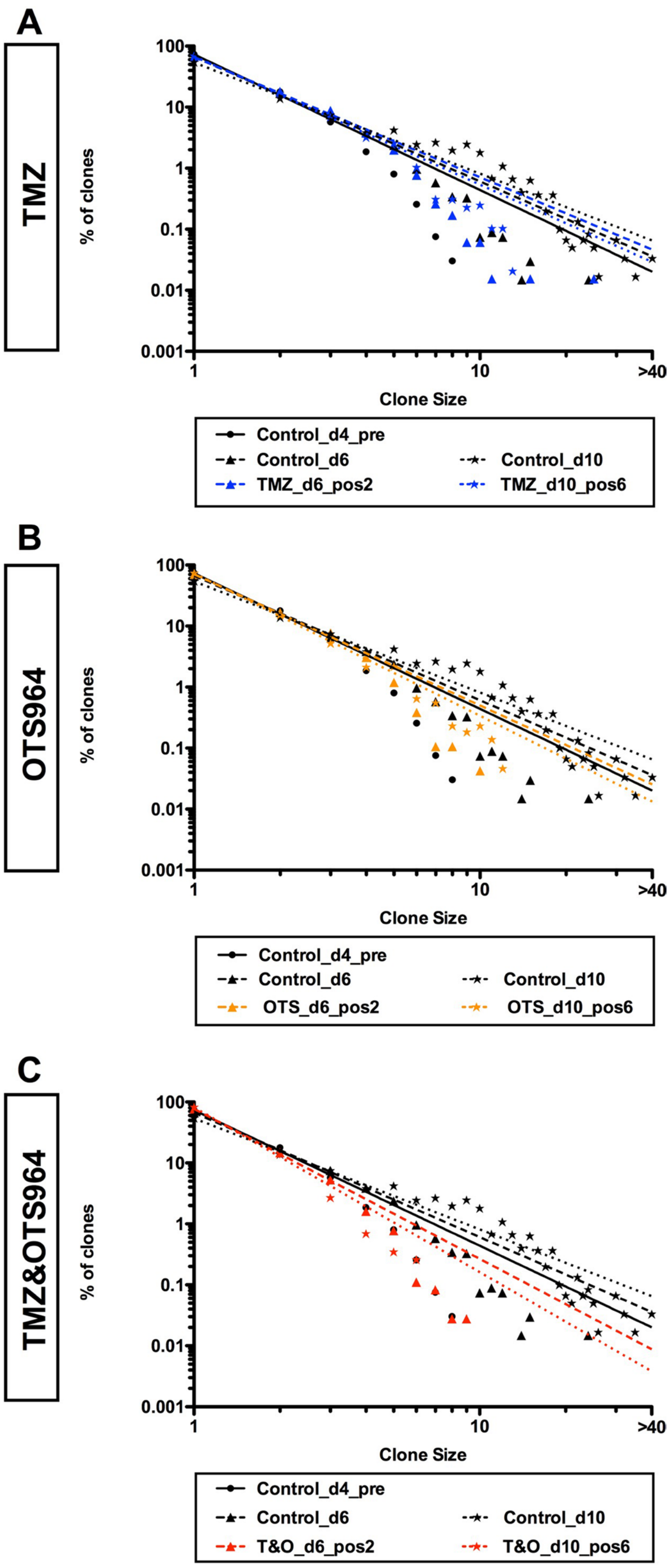

Supplementary Figure 12: U251-GS populations thoroughly protect power-law coded heterogeneity with combined TMZ and OTS964 administration despite early shrinkage. (A–C) The graphs show double logarithmic plots of clone size (the number of cells in each clone) and U251-GS clone numbers for different administration paradigms of 25 µM of TMZ (A, in blue), 300 nM of OTS964 (B, in orange), and combined administration of TMZ and OTS964 (C; T&O, in red) at days 6 and 10 (ex. TMZ_d6_pos2, TMZ_d10_pos6). Every graph shows controls (black labels and lines) at day 4 to represent pre-administration populations (Control_d4_pre), and control populations at days 6 and 10 (Control_d6 and Control_d10), respectively. The circles, triangles and stars represent data at days 4, 6 and 10, respectively. The double logarithmic regression lines use straight, dashed, and dotted lines to represent frequency distributions at days 4, 6 and 10, respectively. The regression lines are based on data from single cell (1-cell) to clones with up to 40 cells. Clones with more than 40 cells are counted as 40 cell clones.
